# Supplementary material for: Radial somatic F‐actin organization affects growth cone dynamics during early neuronal development
Source: EMBO Rep. 2019 Oct 24;20(12):e47743. doi: 10.15252/embr.201947743 (PMC6893363; doi:10.15252/embr.201947743)
Supplement: Supplementary file 1 — Appendix [file EMBR-20-e47743-s001.pdf]

Appendix for

Radial Somatic F-actin Organization Affects Growth Cone Dynamics During Early Neuronal Development

Durga Praveen Meka[1§], Robin Scharrenberg[1§], Bing Zhao[1], Oliver Kobler[2], Theresa König[1], Irina Schaefer[1], Birgit Schwanke[1], Sergei Klykov[3], Melanie Richter[1], Dennis Eggert[4], Sabine Windhorst[5], Carlos G. Dotti[6], Michael R. Kreutz[7,8], Marina Mikhaylova[3], Froylan Calderon de Anda[1*]

[1]RG Neuronal Development, Center for Molecular Neurobiology Hamburg (ZMNH), University Medical Center Hamburg-Eppendorf, 20251 Hamburg, Germany.
[2]Combinatorial Neuroimaging Core Facility (CNI), Leibniz Institute for Neurobiology, 39118 Magdeburg, Germany.
[3]Emmy-Noether Group “Neuronal Protein Transport”, Center for Molecular Neurobiology (ZMNH), University Medical Center Hamburg-Eppendorf, 20251 Hamburg, Germany.
[4]Max Planck Institute for the Structure and Dynamics of Matter, 22761 Hamburg and Heinrich Pette Institute - Leibniz Institute for Experimental Virology, 20251 Hamburg, Germany
[5]Department of Biochemistry and Signal Transduction, University Medical Center Hamburg-Eppendorf, 20246 Hamburg, Germany.
[6]Centro de Biología Molecular ‘Severo Ochoa’, CSIC-UAM, Madrid, Spain.
[7]RG Neuroplasticity, Leibniz Institute for Neurobiology, 39118 Magdeburg, Germany.
[8]Leibniz Guest Group “Dendritic Organelles and Synaptic Function”, Center for Molecular Neurobiology (ZMNH), University Medical Center Hamburg-Eppendorf, 20251 Hamburg, Germany.

[§] Equal contribution
*Lead contact

To whom correspondence should be addressed:
Froylan Calderon de Anda
Email: froylan.calderon@zmnh.uni-hamburg.de
Durga Praveen Meka
Email: praveen.meka@zmnh.uni-hamburg.de
(Tel.): +49 40 7410-56817
(Fax): +49 40 7410-56450

Table of content **page**

Table of content --- **2**
Appendix Figure S1 --- **3**
Appendix Figure S2 --- **5**
Appendix Figure S3 --- **7**
Appendix Figure S4 --- **9**
Appendix Figure S5 --- **11**
Appendix Figure S6 --- **13**
Appendix Figure S7 --- **16**
Appendix Figure S8 --- **18**
Appendix Figure S9 --- **21**
Appendix Figure S10 --- **23**
Appendix Figure S11 --- **25**
Appendix Figure S12 --- **27**
Appendix Figure S13 --- **29**

Appendix Figure S1

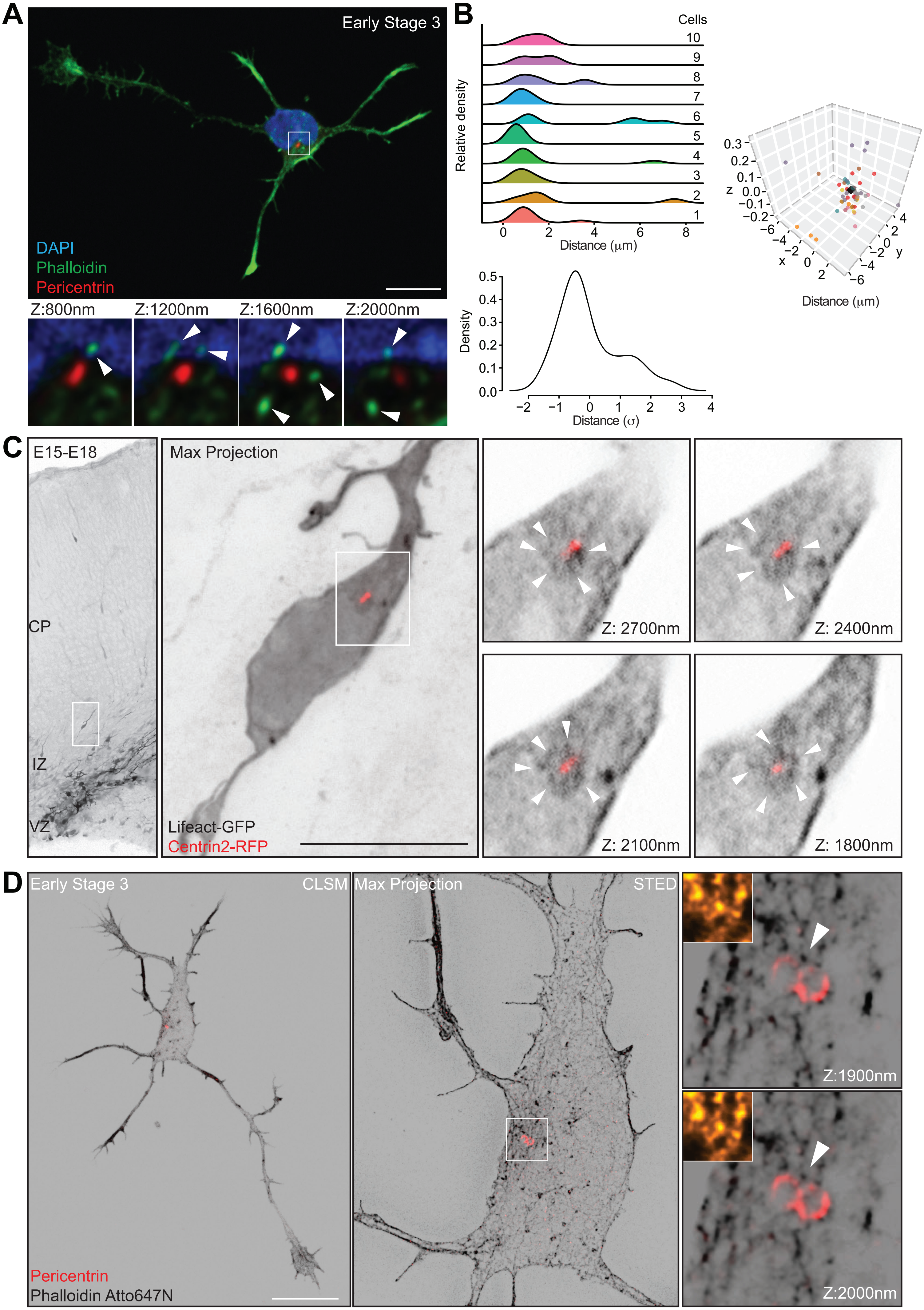

Appendix Figure S1. Confocal microscopy reveals cytosolic F-actin puncta around the centrosome in developing neurons. (A) Early stage 3-hippocampal neuron labelled with phalloidin and Pericentrin antibody. Confocal z-stacks from the inset show F-actin puncta around the centrosome. (**B**) 3D graph shows the distribution of cytosolic F-actin puncta around the centrosome in stage 3 cells. The coordinates of the centrosome and F-actin puncta are obtained from the confocal images. The coordinates of centrosomes from all the cells, indicated as a black cube, is positioned at the center (x, y, z = 0) and the color-coded F-actin puncta are plotted with respect to the position of the centrosome from the respective cell. Ridge plots indicate the density of puncta for individual cells in dependence of distance to the centrosome. Normalization of the distance in each cell reveals a skewed distribution towards the centrosome. Values are centered around the mean and expressed as standard deviations from the mean (z-score). As an overview, puncta coordinates (color-coded for individual cells) are shown in 3D with cells aligned at the centrosome (black spot) n = 10 cells, obtained from at least three different cultures. (**C**) Bipolar cell located in the IZ/CP of the developing cortex expresses Lifeact-GFP and Centrin2-RFP and shows F-actin puncta surrounding the centrosome. (**D**) Confocal (CLSM) and STED microscopic images of an early stage 3 hippocampal neuron. Inset: STED Z-stack images with 100 nm Z-spacing showing F-actin puncta localizing near the centrosome. Insets from arrowheads in Z-stack images show F-actin puncta with F-actin fibers attached. Scale bar: 10 μm.

Appendix Figure S2

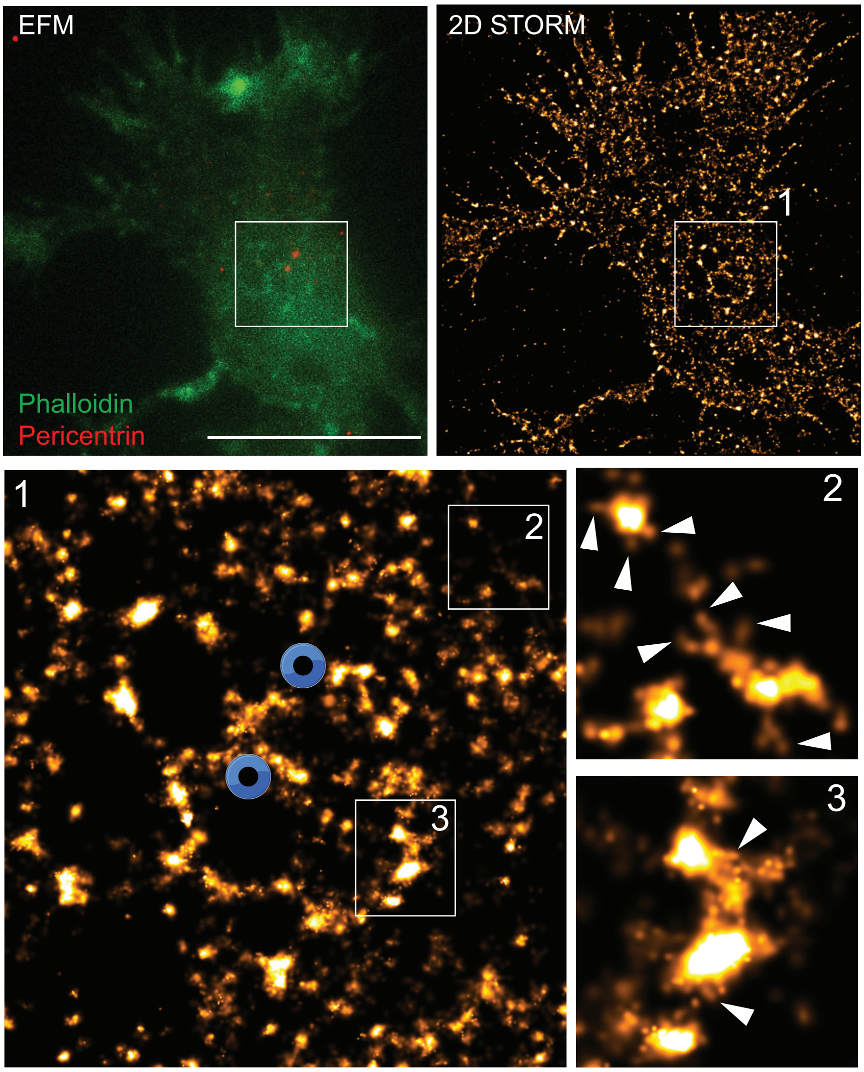

Appendix Figure S2 . Super-resolution microscopy reveals cytosolic F-actin puncta with filaments in developing neurons. Epi-fluorescence (EFM) and SMLM (STORM) images of stage 2 neuron. Inset 1: F-actin puncta near the centrosome (depicted by blue circles) forming a pocket-like structure. Arrowheads from insets 2 and 3 show individual F-actin puncta with F-actin fibers attached. Scale bar: 10 μm.

Appendix Figure S3

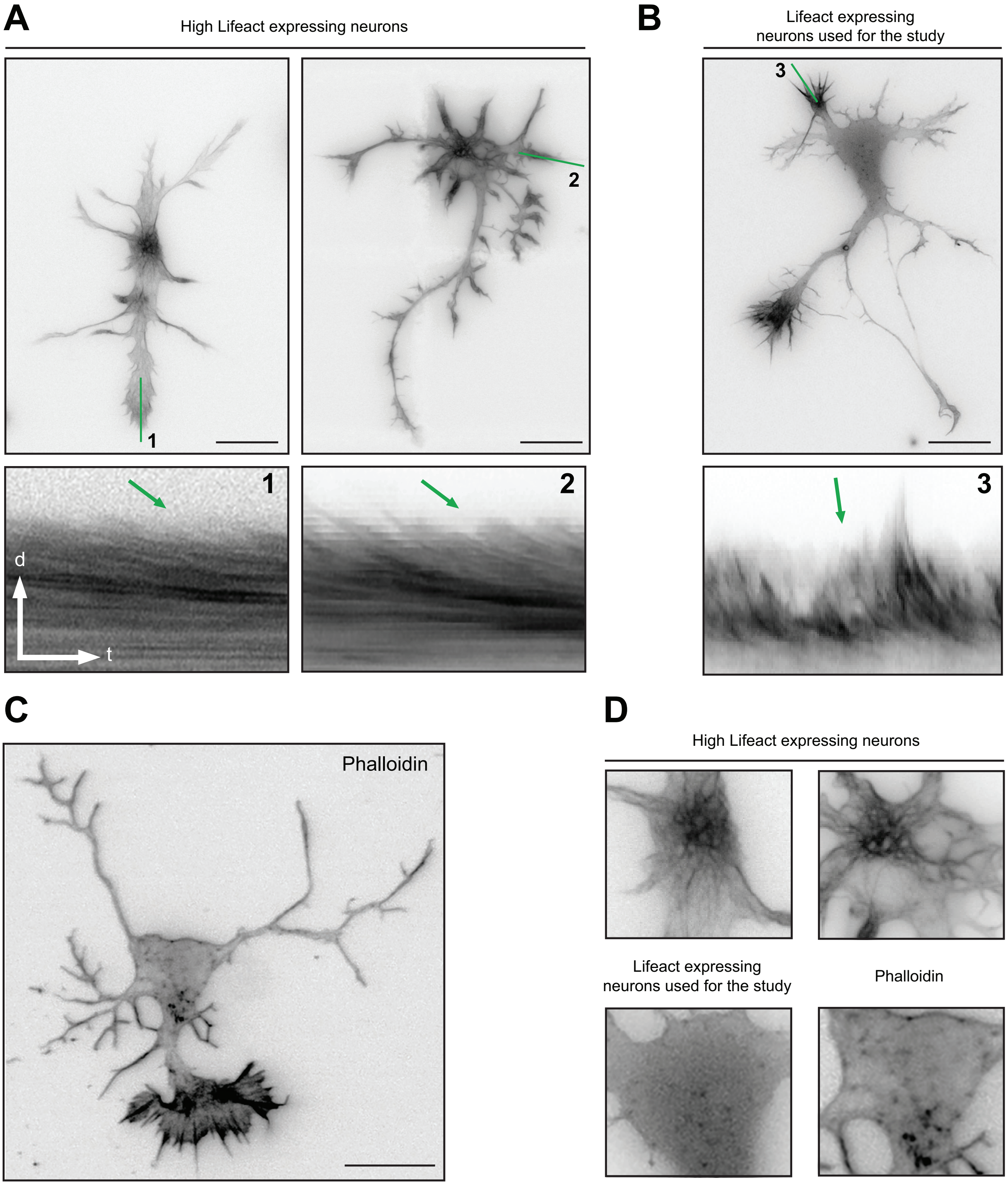

Appendix Figure S3 . Higher expression of Lifeact stabilizes F-actin. (**A**) Cells expressing high levels of Lifeact show less dynamic neurite tips (kymographs) compared with cells expressing Lifeact levels similar to the levels detected with phalloidin staining (B and C, respectively). (**B**) Cells expressing Lifeact and labeling F-actin at comparable levels of Phalloidin staining (**C**) have dynamic neurite tips (Kymograph in bottom panel of **B**). (**D**) Enlarged images of the soma from cells shown in A-C. Scale bar: μm.

Appendix Figure S4

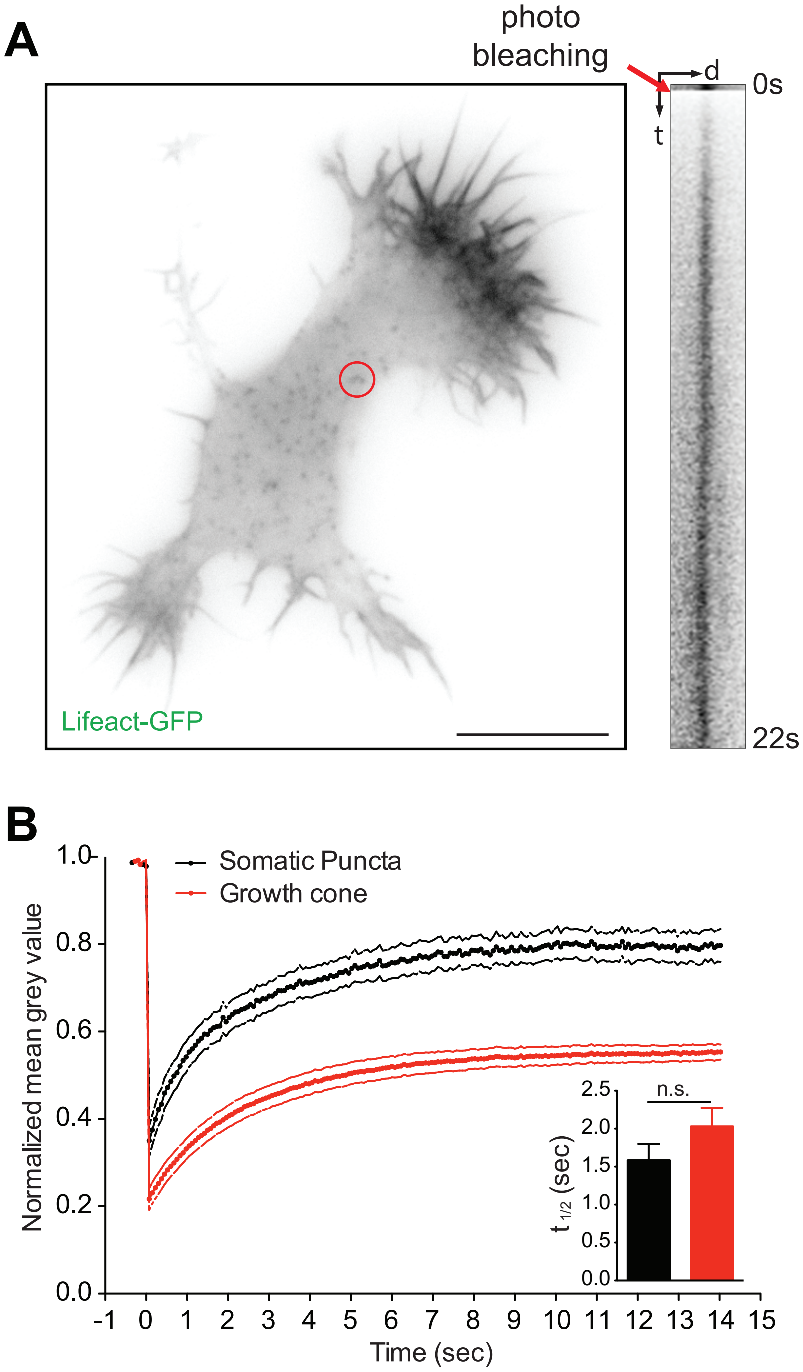

Appendix Figure S4. Somatic F-actin turnover is similar to the turnover in growth cones. (**A**) Lifeact-GFP expressing stage 2 hippocampal neuron photobleached in the soma (region marked by red circle) using 405 nm laser. The kymograph obtained from the photobleached region illustrates fluorescence recovery of the F-actin punctum after photobleaching. (**B**) Time course of the normalized fluorescence intensity in FRAPed region in the somatic and growth cone regions of Lifeact-GFP expressing neurons. Half-time (t ½) values were shown in the inset. t ½ (in sec) for somatic puncta = 1.584 ± 0.2151, growth cones = 2.030 ± 0.2422. n.s = not significant, $p = 0.2224$ by unpaired Student's t-test. Mean ± SEM; n = 9 cells for somatic puncta and n = 15 cells for growth cones. Cells were obtained from at least two different cultures. Scale bar: 10 μm.

Appendix Figure S5

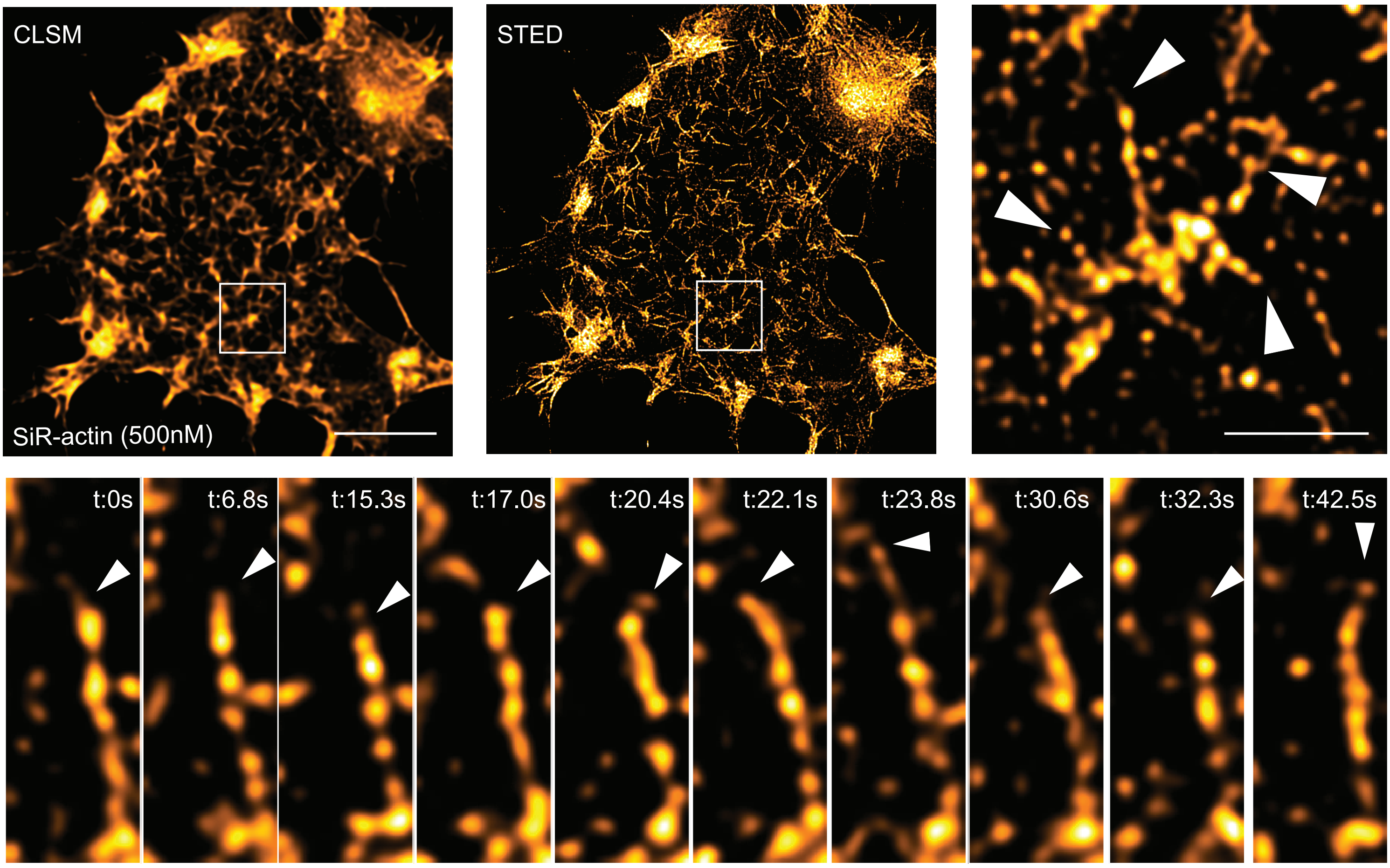

Appendix Figure S5. Super-resolution microscopy reveals that higher concentration of SiR-actin (500nM) induced the formation of somatic F-actin fibers. With a higher concentration of SiR-actin, the somatic F-actin puncta have longer F-actin fibers attached to them with less activity (lower panel arrowheads), compared with the lower SiR-actin concentration used (Fig. 1E). Scale bar: 2 μm and 0.5 μm (insets).

Appendix Figure 6

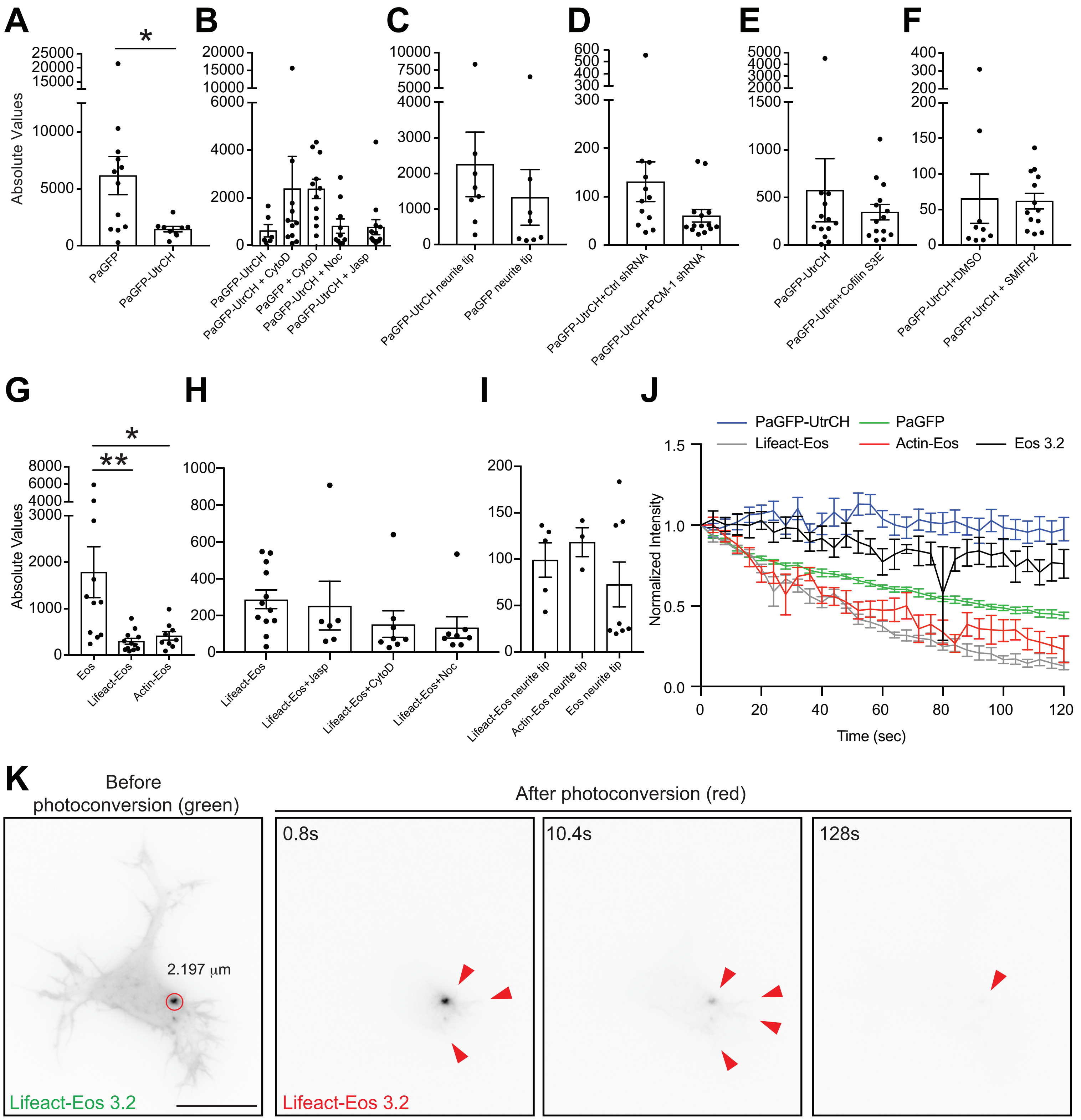

Appendix Figure S6. Characterization of Photoactivatable and Photoconvertible probes used in the study. (**A - F**) Graphs show absolute fluorescence values of photoactivated signal in the rat hippocampal or mouse cortical neurons expressing PaGFP or PaGFP-UtrCH that are used for analysis in this study. Values are measured from the first frame of acquisition in the regions of 405 nm laser illumination. (**A**) Absolute fluorescence values of PaGFP = 6156 ± 1673, PaGFP-UtrCH = 1458 ± 235.6. *p = 0.0267 by Student's t-test. (Data related to Fig. 2). (**B**) Absolute fluorescence values of PaGFP-UtrCH = 613.8 ± 260.8, PaGFP-UtrCH + CytoD = 2378 ± 1356, PaGFP + CytoD = 2369 ± 406.6, PaGFP-UtrCH + Noc = 806.8 ± 300.5, PaGFP-UtrCH + Jasp = 762.2 ± 317.0. p = 0.2030 by one-way ANOVA (Data related to Fig. 5 F, G; Fig. EV3 D, E and Appendix Fig. S8 C, D). (**C**) Absolute fluorescence values of PaGFP neurite tip = 1330 ± 780.7, PaGFP-UtrCH neurite tip = 2255 ± 906.7. p = 0.4524 by Student's t-test (Data related to Fig. EV4 A-C). (**D**) Absolute fluorescence values of PaGFP-UtrCH + Ctrl shRNA = 130.7 ± 41.19, PaGFP-UtrCH + PCM-1 shRNA = 60.33 ± 13.08. p = 0.0955 by Student's t-test (data related to Fig. 6 G-I). (**E**) Absolute fluorescence values of PaGFP-UtrCH = 575.7 ± 331.5, PaGFP-UtrCH + Cofilin-S3E = 345.9 ± 81.49. p = 0.4926 by Student's t-test (Data related to Appendix Fig. S9 A-C). (**F**) Absolute fluorescence values of PaGFP-UtrCH + DMSO = 65.4 ± 34.55, PaGFP-UtrCH + SMIFH2 = 61.99 ± 10.97. p = 0.9144 by Student's t-test. (Data related to Fig. 7 D-F). (**G - I**) Graphs shows absolute fluorescence values of photoconverted signal in the rat hippocampal neurons expressing mEos3.2, Lifeact-mEos3.2 or Actin-Eos4b that are used for analysis in this study. Values are measured from the first frame of acquisition in the regions of 405 nm laser illumination. (**G**) Absolute fluorescence values of mEos3.2 = 1783 ± 545.5, Lifeact-mEos3.2 = 298.5 ± 62.07. Actin-mEos4b = 415.5 ± 91.43. p=0.0050 by one-way ANOVA, post hoc Bonferroni's test, ** p<0.01, *p<0.05. (Data related to Fig. 3 and Fig. EV2). (**H**) Absolute fluorescence values of Lifeact-mEos3.2 = 288.1 ± 50.86; LifeactmEos3.2+ Jasp = 253.5 ± 132.4, Lifeact-mEos3.2+ CytoD = 153.2 ± 71.80, LifeactmEos3.2+ Noc = 135.1 ± 57.77. p=0.3444 by one-way ANOVA. (Data related to Fig. EV3 F, G; Fig. EV5 E, F and Appendix Fig. S8 E, F). (**I**) Absolute fluorescence values of Lifeact-mEos3.2 neurite tip = 99.08 ± 18.62, Actin-mEos4b neurite tip = 118.3 ± 15.63, mEos3.2 neurite tip = 76.66 ± 24.20. p = 0.4612 by one-way ANOVA. (Data related to Appendix Fig. S7). All graphs in A – I: Data is represented as Mean ± SEM. (**J**) In order to account for photobleaching of our probes, photoactivation/photoconversion of fixed samples were performed. Graphs shows fluorescence bleach of photoactivated or photoconverted signal in PFA-fixed rat hippocampal neurons expressing PaGFP, PaGFP-UtrCH, mEos3.2, Lifeact-mEos3.2 or Actin-Eos4b. Mean ± SEM; n = 10 each for PaGFP-UtrCH and PaGFP

cells, n = 5 for mEos3.2 cells, n = 6 for Lifeact-mEos3.2 14 cells and n = 8 for Actin-Eos4b cells. Cells were obtained from at least two different cultures. (**K**) Lifeact-mEos3.2-expressing stage 2 neuron photoconverted in the soma with 405 nm laser (red circle with a dimeter of 2.197 µm). Cell before (green signal) and after photoconversion (red signal). Scale bar: 10 µm.

Appendix Figure S7

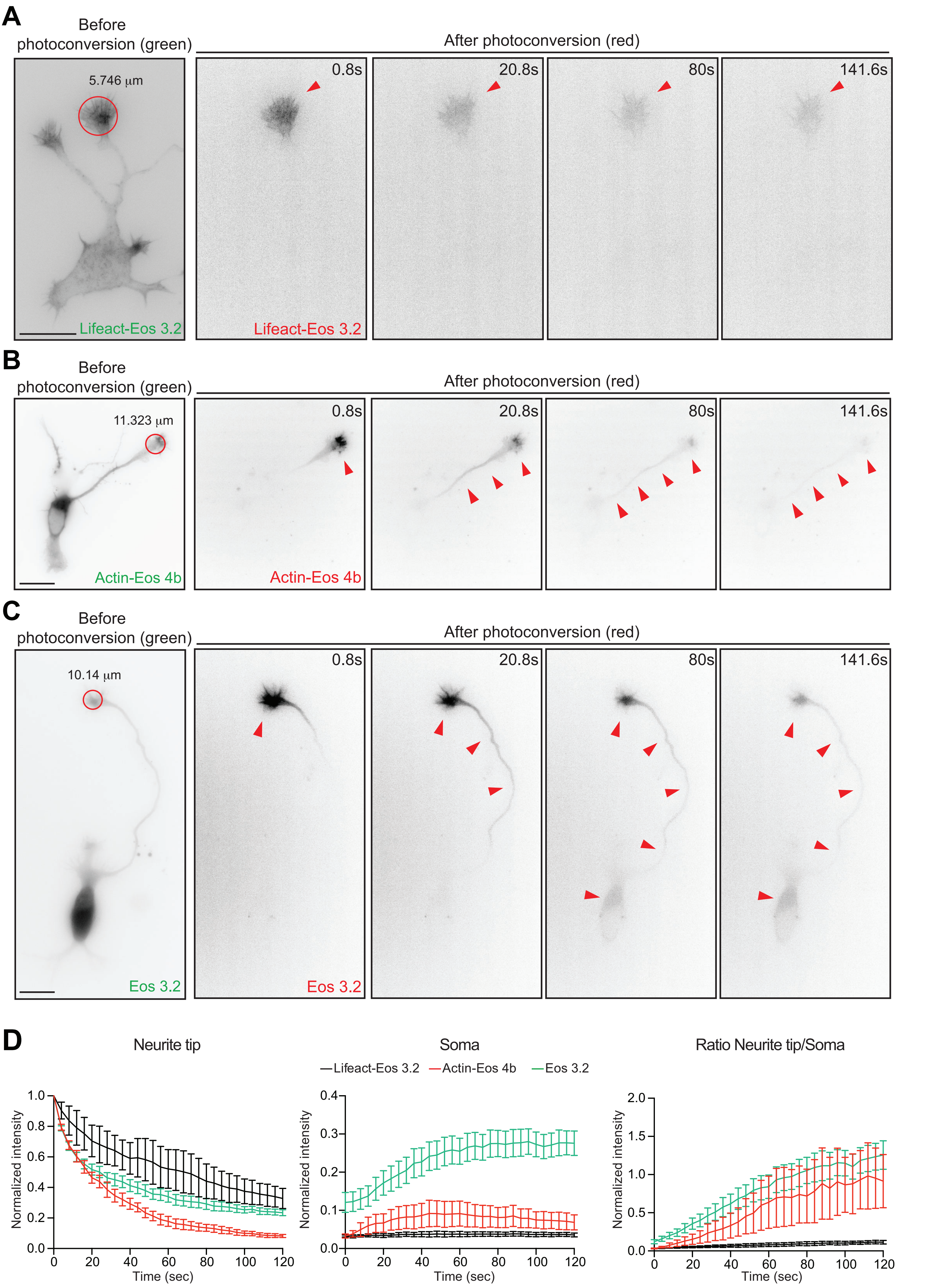

Appendix Figure S7. Photoconverted Lifeact-mEos3.2 in neurite tips do not translocate towards the cell body. (**A**) Lifeact-mEos3.2 expressing cell before (green) and after photoconversion (red) at the neurite tip with 405 nm laser (red circle with a diameter of 5.746 µm). Red arrowheads point the reach of the photoconverted signal over time. (**B**) Actin-Eos4b expressing cell before (green) and after photoconversion (red) at the neurite tip with 405 nm laser (red circle with a diameter of 11.323 µm). Red arrowheads point the reach of the photoconverted signal over time. (**C**) Eos3.2 expressing cell before (green) and after photoconversion (red) at the neurite tip with 405 nm laser (red circle with a diameter of 10.14 µm). Red arrowheads point the reach of the photoconverted signal over time. (**D**) Left panel: normalized intensity values in the photoconverted area (neurite tips) of Lifeact-mEos3.2, Actin-mEos4b or mEos3.2 expressing cells. Middle panel: photoconverted signal in the soma over time relative to the average initial signal from illuminated area for Lifeact-mEos3.2, Actin-mEos4b and mEos3.2 expressing cells. Right panel: neurite tip to soma photoconverted signal intensity ratio of Lifeact-mEos3.2, Actin-mEos4b and mEos3.2 expressing cells. Mean ± SEM; n = 3 cells for Lifeact-mEos3.2, n = 3 cells for Actin-mEos4b, and n = 7 cells for mEos3.2 from two different cultures. Scale bar: 10 µm

Appendix Figure S8

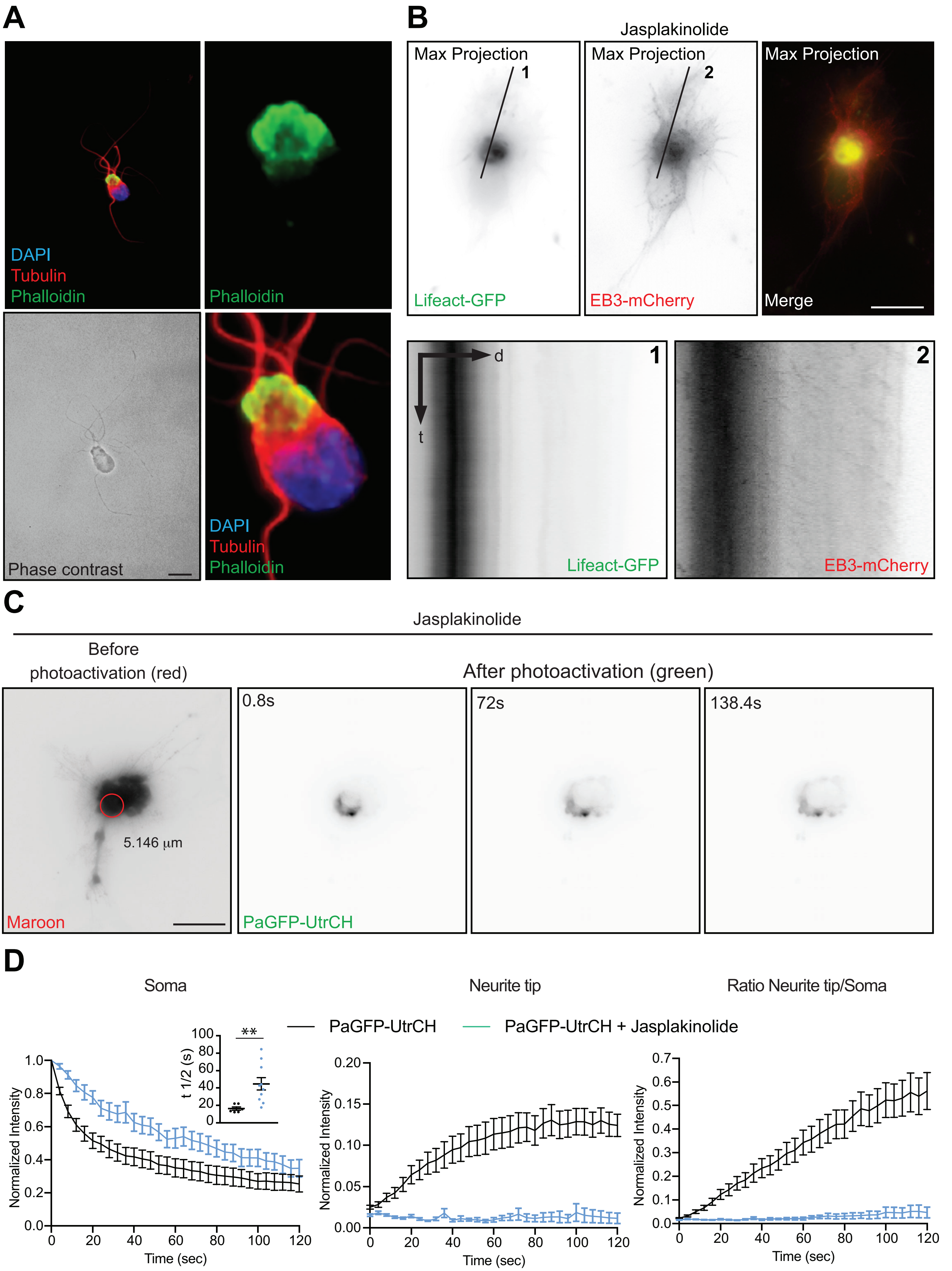

Appendix Figure S8

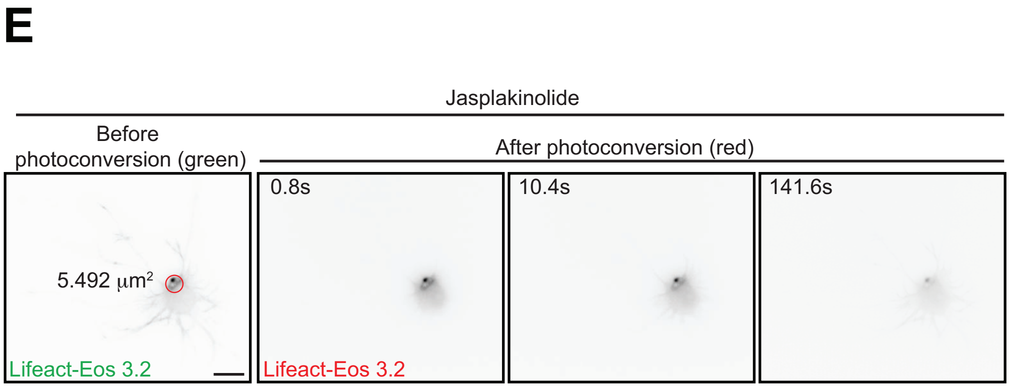

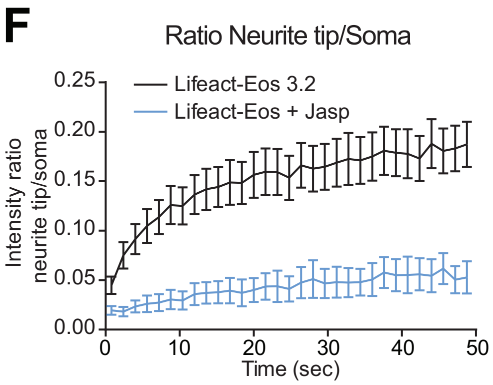

Appendix Figure S8. Jasplakinolide treatment blocks F-actin at the soma. (**A**) Jasplakinolide promotes the formation of a F-actin pocket-like structure in the soma (91.01% of 189 cells from at least three different cultures). (**B**) Neuron treated with Jasplakinolide shows the formation of a F-actin structure at the region of higher EB3 density. Kymograph shows that this F-actin structure is not dynamic. (**C-E**) Jasplakinolide treatment (300 nM for 1.5 hrs.) precludes translocation of photoactivated PaGFP-UtrCH or photoconverted Lifeact-mEos3.2 signal from soma to the cell periphery. (**C**) PaGFP-UtrCH and mMaroon1 co-transfected neuron treated with Jasplakinolide was illuminated in the soma using 405 nm laser (red circle with a dimeter of 5.146 µm). The distribution of green signal shows the effect of F-actin polymerization on the movement of PaGFP-UtrCH from the soma to the periphery. (**D**) Left panel: normalized intensity values in the photoactivated area (soma) of untreated, Jasplakinolide treated PaGFP-UtrCH expressing cells. Inset graph: half-time (t ½) values in sec for Untreated cells = 16.29 ± 1.884 (n =6), Jasplakinolide treated cells = 44.78 ± 7.095 (n = 10). **p = 0.009 by unpaired Student's t-test. Middle panel: photoconverted signal in the neurite tip over time relative to the average initial signal from illuminated area for untreated, Jasplakinolide treated PaGFP-UtrCH expressing cells. Right panel: Neurite tip to soma intensity ratio of photoactivated untreated, Jasplakinolide treated PaGFP-UtrCH expressing cells. All panels: Mean ± SEM; n = 6 for untreated cells, n = 12 for Jasplakinolide treated PaGFP-UtrCh cells, from at least 2 different cultures. Experiments shown in Fig. 5 F, G; Fig. EV3 D, E and Appendix Fig. S8 C, D were done at the same time, therefore the same Control data (Untreated PaGFP-UtrCH) is used for comparison. (**E**) Lifeact-mEos3.2 expressing cell treated with Jasplakinolide and photoconverted in the soma with 405 nm laser (red circle with a dimeter of 5.239 µm). Cell before (green) and after (red) photoconversion. (**F**) Neurite tip to soma intensity ratio of photoconverted signal in untreated, Jasplakinolide treated Lifeact-Eos3.2 cells. Experiments shown in Fig. EV3 F, G; Fig. EV5 E, F and Appendix Fig. S8 E, F were done at the same time, therefore the same Control data (Untreated Lifeact-Eos3.2) is used for comparison. All panels: Mean ± SEM; n = 12 for untreated cells, n = 9 for Jasplakinolide treated Lifeact-Eos3.2 cells from at least 3 different cultures. Scale bar: 10 µm

Appendix Figure S9

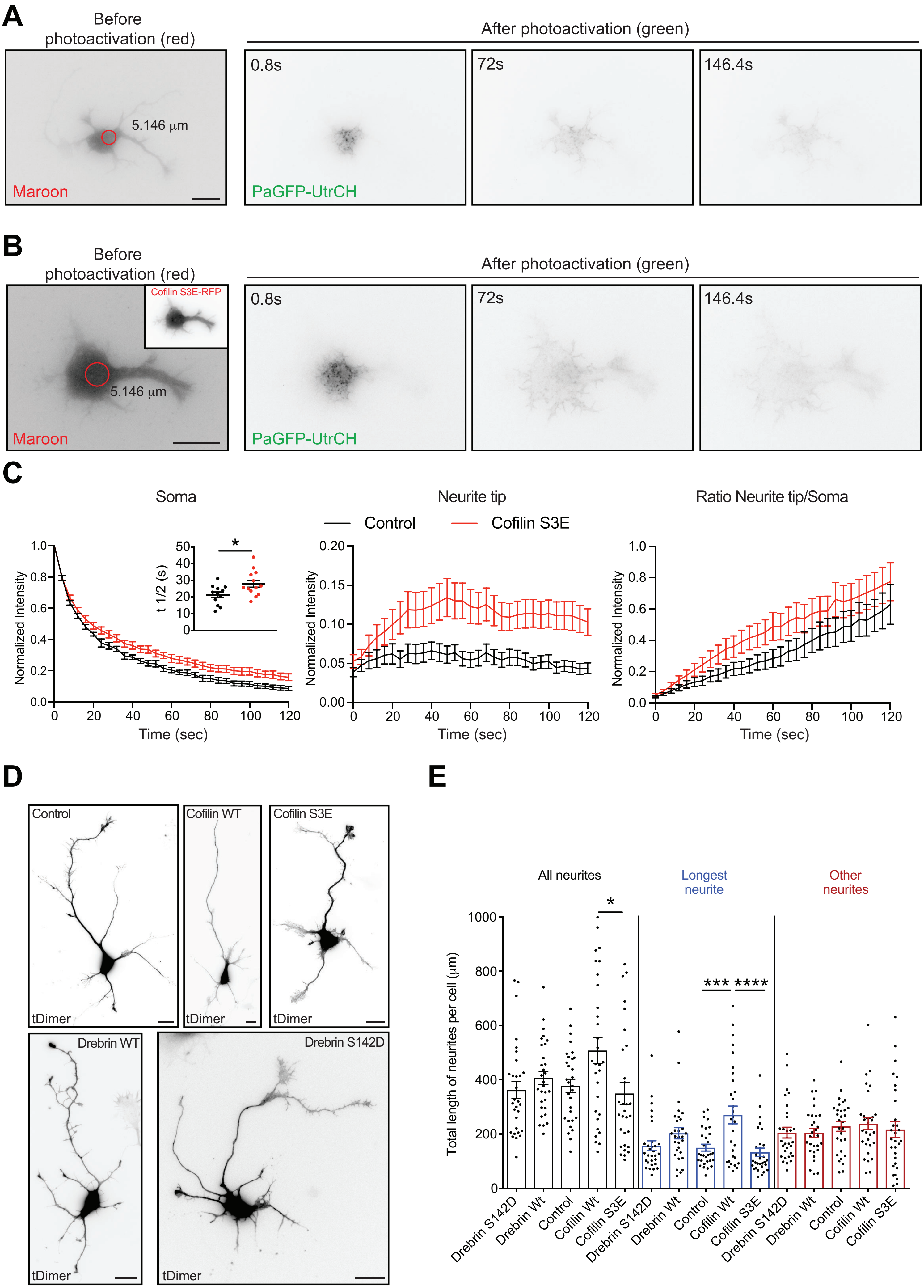

Appendix Figure S9. Reducing the dynamics of F-actin, by overexpression of Cofilin phospho mutants affects soma to neurite F-actin translocation and neurite growth (**A, B**) DIV1 Rat hippocampal neurons before (red; Maroon) and after photoactivation (green; PaGFP-UtrCH, A; PaGFP-UtrCH+Cofilin-S3E-RFP, B) in the soma with 405 nm laser (red circle with a diameter of 5.146 µm). (**C**) Left panel: normalized intensity values in the photoactivated area of PaGFP-UtrCH expressing cells. Inset graph: half-time (t ½) values in sec for PaGFP-UtrCH = 21.45 ± 1.686, PaGFP-UtrCH + Cofilin-S3E-RFP = 28,11 ± 2,087. p = 0.0220 by unpaired Student's t-test, *p<0.05. Middle panel: photoconverted signal in neurite tips over time relative to the average initial signal from illuminated area for PaGFP-UtrCH expressing cells. Right panel: Neurite tip to soma photoactivated signal intensity ratio of PaGFP-UtrCH expressing cells. All panels: Mean ± SEM; n = 12 cells for PaGFP-UtrCH and n = 13 cells for PaGFP-UtrCH + Cofilin-S3E-RFP, from at least two different cultures. (**D**-**E**) Cofilin- phospho-mimetic overexpression normalizes the neurite elongation phenotype of its wildtype counterpart, Drebrin-Wt and phospho-mimetic showed no significant differences (**D**) DIV3 rat hippocampal primary neurons expressing tDimer alone (Control) or together with Cofilin-WT, Cofilin-S3E, Drebrin-WT, and Drebrin-S142D. (**E**) Total length of neurites per cell (in µm). Length of all neurites in each condition: Control = 337.8 ± 24.68, Cofilin-Wt = 507.8 ± 48.11, Cofilin-S3E= 349.5 ± 40.19, Drebrin-Wt = 407.1 ± 24.37, Drebrin-S142D = 362.3 ± 31.29. Length of longest neurite in neurites in each condition: Control = 149.8 ± 12.47, Cofilin-Wt= 270.1 ± 32.76, Cofilin-S3E= 132.6 ± 15.53, Drebrin-Wt = 202.8 ± 20.56, Drebrin-S142D = 157.3 ± 17.72. Length of other neurites in in each condition: Control = 228.0 ± 17.94, Cofilin-Wt = 237.7 ± 22.06, Cofilin-S3E= 216.9 ± 29.02, Drebrin-Wt = 204.3 ±16.44, Drebrin-S142D = 205.0 ± 19.81. p<0.0001 by one-way ANOVA, post hoc Tukey test, ****p<0.0001, ***p<0.001, *p<0.05. Mean ± SEM; n = 30 cells for each group from at least two different cultures. Scale bar: 10 µm.

Appendix Figure S10

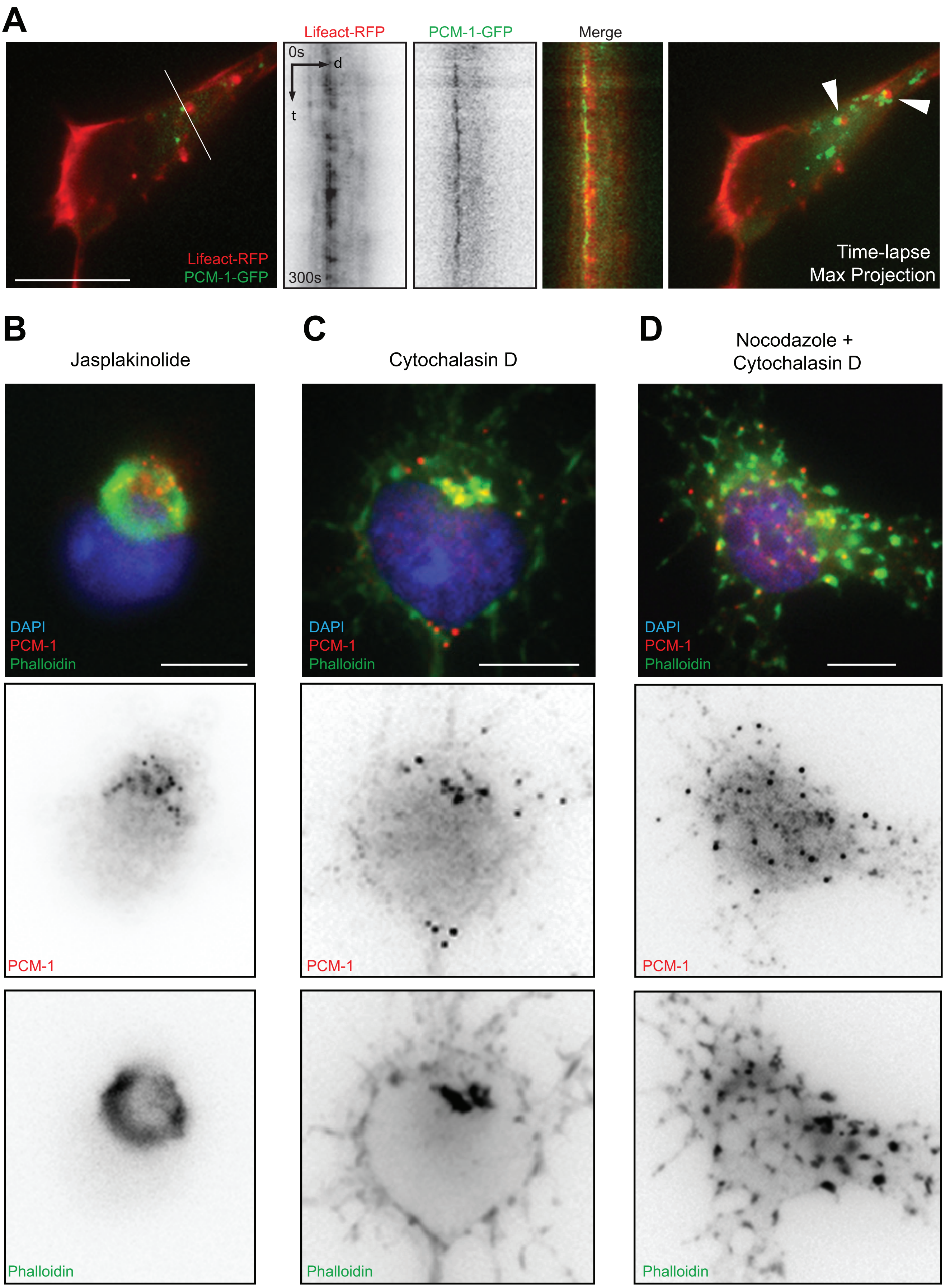

Appendix Figure S10. PCM-1 intermingles with F-actin puncta. (**A**) PCM-1-GFP expressing-cells show close association of PCM-1 and F-actin puncta. Kymographs obtained from white-line and arrowheads in Max Projection of the time-lapse show close association of F-actin and PCM-1. (**B**) Jasplaklinolide treatment (500 nM for 4 hrs.) induces the formation of a somatic F-actin "ring" structure accompanied by PCM-1 particles**.** (**C**) Cytochalasin D (1μM for 3 hrs.) treatment leads to the formation of somatic F-actin clusters accompanied by PCM-1 particles. (**D**) Nocodazole (7μM) and Cytochalasin D (1μM) treatment (for 3 hrs.) disperses somatic F-actin clusters which are accompanied by PCM-1 particles. Cells were stained with PCM-1 antibody, phalloidin (labels F-actin) and DAPI (stains nucleus). Scale bar: 10 μm (A), 5 μm (B-D).

Appendix Figure S11

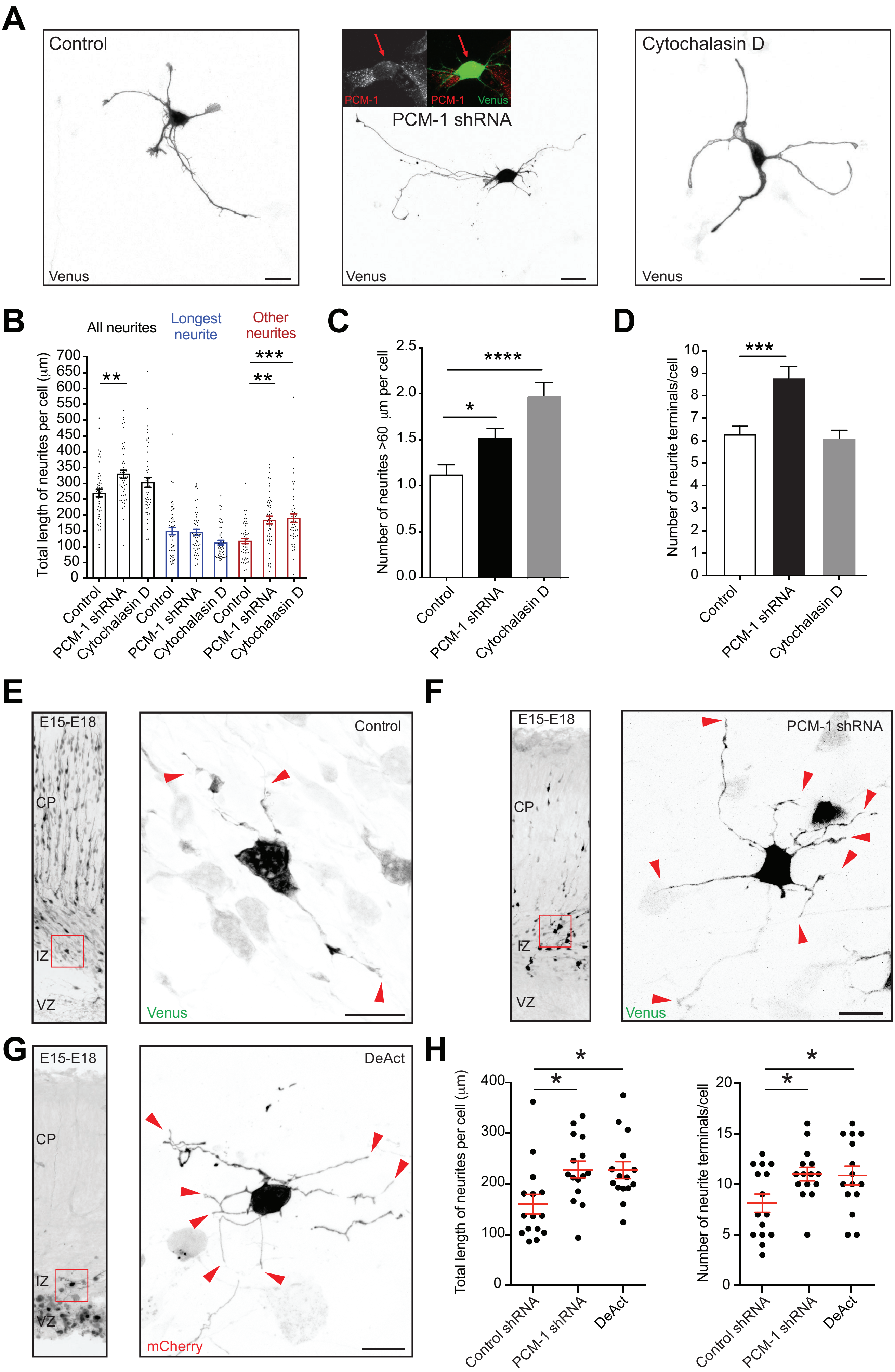

Appendix Figure S11. PCM-1 downregulation affects neurite elongation. (**A**) DIV 2 cortical neurons from E17 mouse embryos that were in utero electroporated at E15 with Control or PCM-1-shRNA or cells from the Control group were treated with 1μM Cytochalasin D 24 h after plating for Cytochalasin D condition. Venus was co-electroporated in all the conditions as a transfection marker. Anti-PCM-1 antibody staining shown in the PCM-1 shRNA insert confirms efficient knockdown of PCM-1 in the Venus transfected cell (pointed with a red arrow). (**B**-**D**) Cytochalasin D and PCM-1 down-regulation boost similarly neurite outgrowth. (**B**) Total length of neurites per cell (in µm). Length of all neurites in Control condition = 269.5 ± 12.18, PCM-1-shRNA condition = 329.5 ± 12.55, cytochalasin D condition = 303.3 ± 15.38. Length of longest neurite in Control condition = 117.8 ± 8.054, PCM-1-shRNA condition = 184.0 ± 12.05, cytochalasin D condition = 190.3 ± 13.06. Length of other neurites in Control condition = 149.5 ± 11.58, PCM-1-shRNA condition = 145.4 ± 9.357, cytochalasin D condition = 113.0 ± 7.318. $p<0.0001$ by one-way ANOVA, post hoc Tukey test, ***$p<0.001$, **$p<0.01$, n.s = not significant. (**C**) Number of neurites greater than 60 µm per cell in Control condition = 1.120 ± 0.1093, PCM-1-shRNA condition = 1.520 ± 0.1040, cytochalasin D condition = 1.980 ± 0.1414; $p<0.0001$ by one-way ANOVA, post hoc Dunnett's test, ****$p<0.0001$, *$p<0.05$. (**D**) Quantification showing number of neurite tips per cell in Control condition = 6.30 ± 0.3571, PCM-1-shRNA condition = 8.780 ± 0.52, cytochalasin D condition = 6.10 ± 0.3651; $p<0.0001$ by one-way ANOVA, post hoc Dunnett's test, ***$p<0.001$. Mean ± SEM; n = 50 cells for each group from at least three different cultures (for data shown in **B**, **C** and **D)**. (**E**) PCM-1 downregulation or actin depolymerization (via DeAct expression) result in neurite elongation and increase in number of neurite terminals *in vivo*. Inset: Multipolar cells in the intermediate zone (IZ) expressing control shRNA, PCM-1 shRNA or DeAct plasmids together with Venus or mCherry. (**H**) Left panel: Total length of neurites per cell in control condition = 160 ± 19.37, PCM-1 shRNA = 228.5 ± 16.82, and DeAct expressing cells = 227 ± 16.71. p = 0.0123 by one-way ANOVA, post hoc Dunnett's test. *$p<0.05$. Right panel: Number of neurite terminals per cell in control condition = 8.133 ± 0.88, PCM-1 shRNA = 11 ± 0.66, and DeAct expressing cells = 10.87 ± 0.93. p = 0.0321 by one-way ANOVA, post hoc Dunnett's test. *$p<0.05$. Mean ± SEM; n = 15 cells from three brains in each group. Scale bar: 10 μm.

Appendix Figure S12

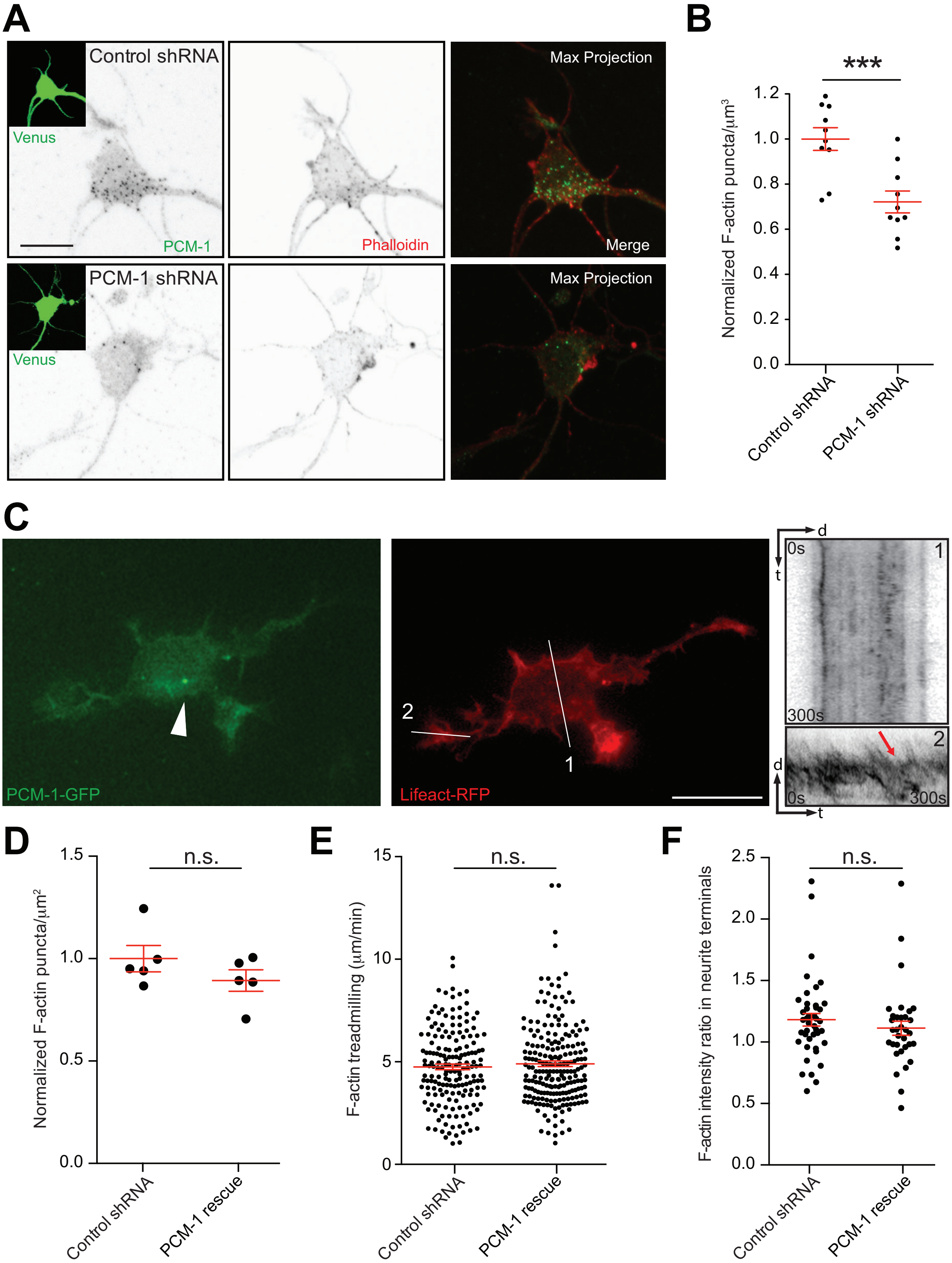

Appendix Figure S12. PCM-1 down-regulation affects somatic F-actin puncta and is reversed by a PCM-1 shRNA resistant plasmid. (**A, B**) PCM-1 down-regulation decreased the density of somatic F-actin puncta detected with phalloidin staining of stage 2 cells from control condition = 1.000 ± 0.0500, PCM-1 shRNA condition = 0.7212 ± 0.0485; p = 0.0008 by unpaired Student's t-test. Mean ± SEM; n = 10 cells each for control and PCM-1 shRNA groups from at least three different cultures. (**C**) DIV 1 cortical neuron from E17 mouse embryos *in utero* electroporated at E15 with PCM-1 shRNA together with Lifeact-RFP and Chicken-PCM-1-GFP (PCM-1 shRNA resistant plasmid). Arrowhead points to PCM-1-GFP puncta. Kymographs obtained from white lines marked as 1 (for soma) and 2 (for neurite tip). (**D-F**) Quantifications from control (control shRNA + Lifeact-RFP) and PCM-1 rescue (PCM-1 shRNA + Chicken-PCM-1-GFP + Lifeact RFP) conditions: (**D**) Density of somatic F-actin puncta of stage 2 cells from control condition = 1.000 ± 0.06468, PCM-1 rescue condition = 0.8937 ± 0.05249; p = 0.2378 by unpaired Student's t-test. Mean ± SEM; n = 5 cells each for control and PCM-1 rescue groups. Cells were obtained from at least two different cultures. (**E**) F-actin treadmilling speed (in µm/min) in the neurite tips of stage 2 cells from control condition = 4.753 ± 0.1390, PCM-1 rescue condition = 4.905 ± 0.1346, p = 0.4357 by unpaired Student's t-test. Mean ± SEM; n = 9 cells each for control and PCM-1 rescue groups. Cells were obtained from at least two different cultures. (**F**) F-actin intensity ratio in the neurite tips of stage 2 cells from control condition = 1.182 ± 0.05147, PCM-1 rescue condition = 1.113 ± 0.05770. n.s = not significant (p = 0.3746) by unpaired Student's t-test. Mean ± SEM; n = 9 cells for control and n = 8 cells for PCM-1 rescue from at least two different cultures. Scale bar: 10 µm.

Appendix Figure S13

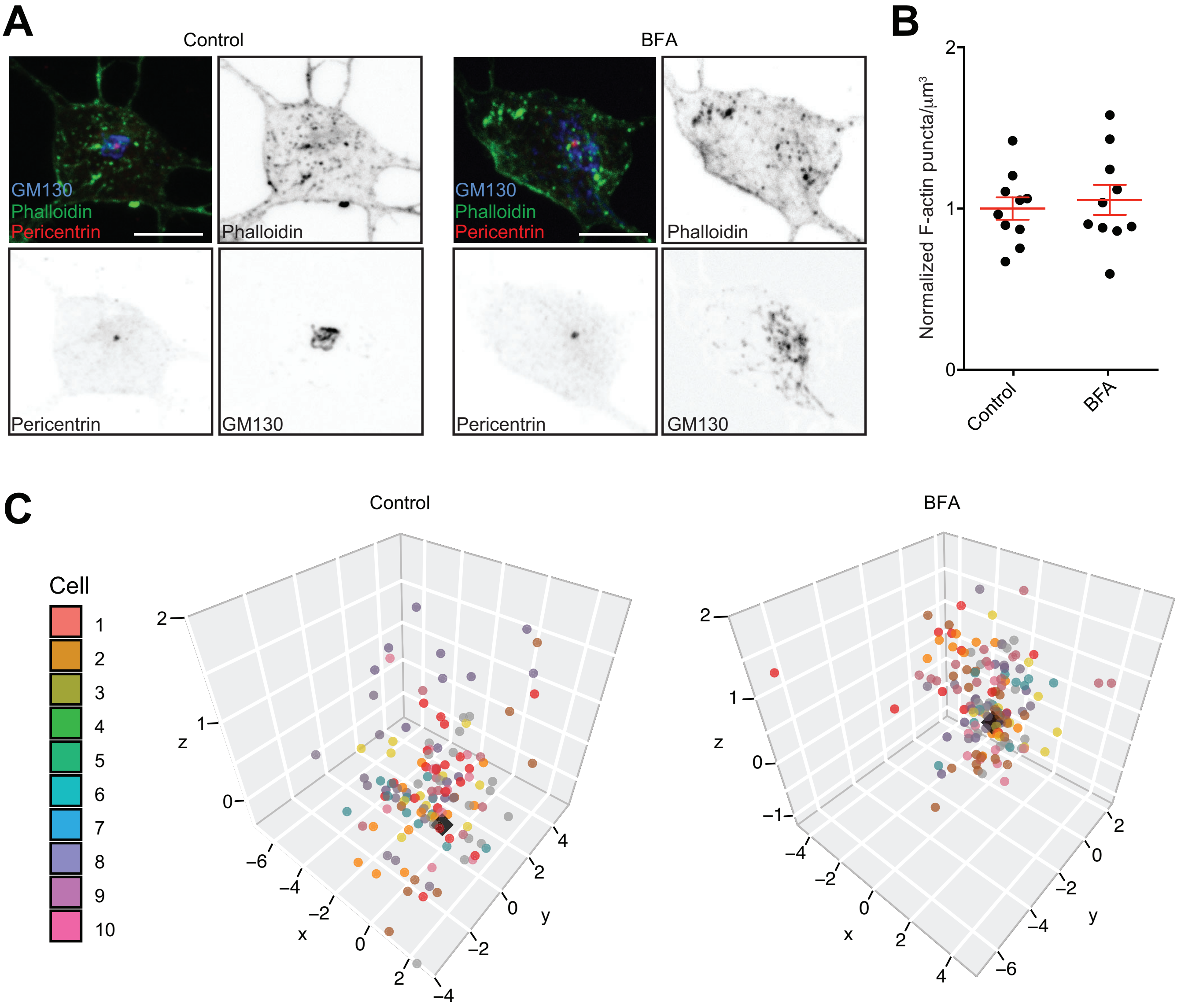

Appendix Figure S13 . Membrane (Golgi, ER, endosomal and lysosomal) disruption did not affect F-actin puncta inside the cell.

(**A**-**C**) Control and BFA (1mg/ml for 12 hrs.) treated DIV1 rat hippocampal neurons showed similar F-actin distribution around the centrosome. (**A**) Confocal images of Control and BFA treated cells are labelled for F-actin (Phalloidin), Centrosome (Pericentrin) and membranes (GM130). (**B**) Quantification show no differences in the number of normalized F-actin puncta in Control and BFA treated cells. Normalized F-actin puncta per μm^3 in Control cells = 1.00 ± 0.069, BFA treated cells = 1.054 ± 0.093. p = 0.651 by Student's t-test. Mean ± SEM; n = 10 cells per group from two independent experiments. (**C**) 3D graphs show the distribution of F-actin puncta around the centrosome in Control and BFA treated cells. The coordinates of the centrosome and F-actin puncta are obtained from the confocal images. The coordinates of centrosomes from all the cells, indicated as a black cube, is positioned at the center (x, y, z = 0) and the color-coded F-actin puncta are plotted with respect to the position of the centrosome from the respective cell. Scale bar: 5 μm.
