# Supplementary material for: Radial somatic F‐actin organization affects growth cone dynamics during early neuronal development
Source: EMBO Rep. 2019 Oct 24;20(12):e47743. doi: 10.15252/embr.201947743 (PMC6893363; doi:10.15252/embr.201947743)
Supplement: Supplementary file 2 — Expanded View Figures PDF [file EMBR-20-e47743-s002.pdf]

# Expanded View Figures

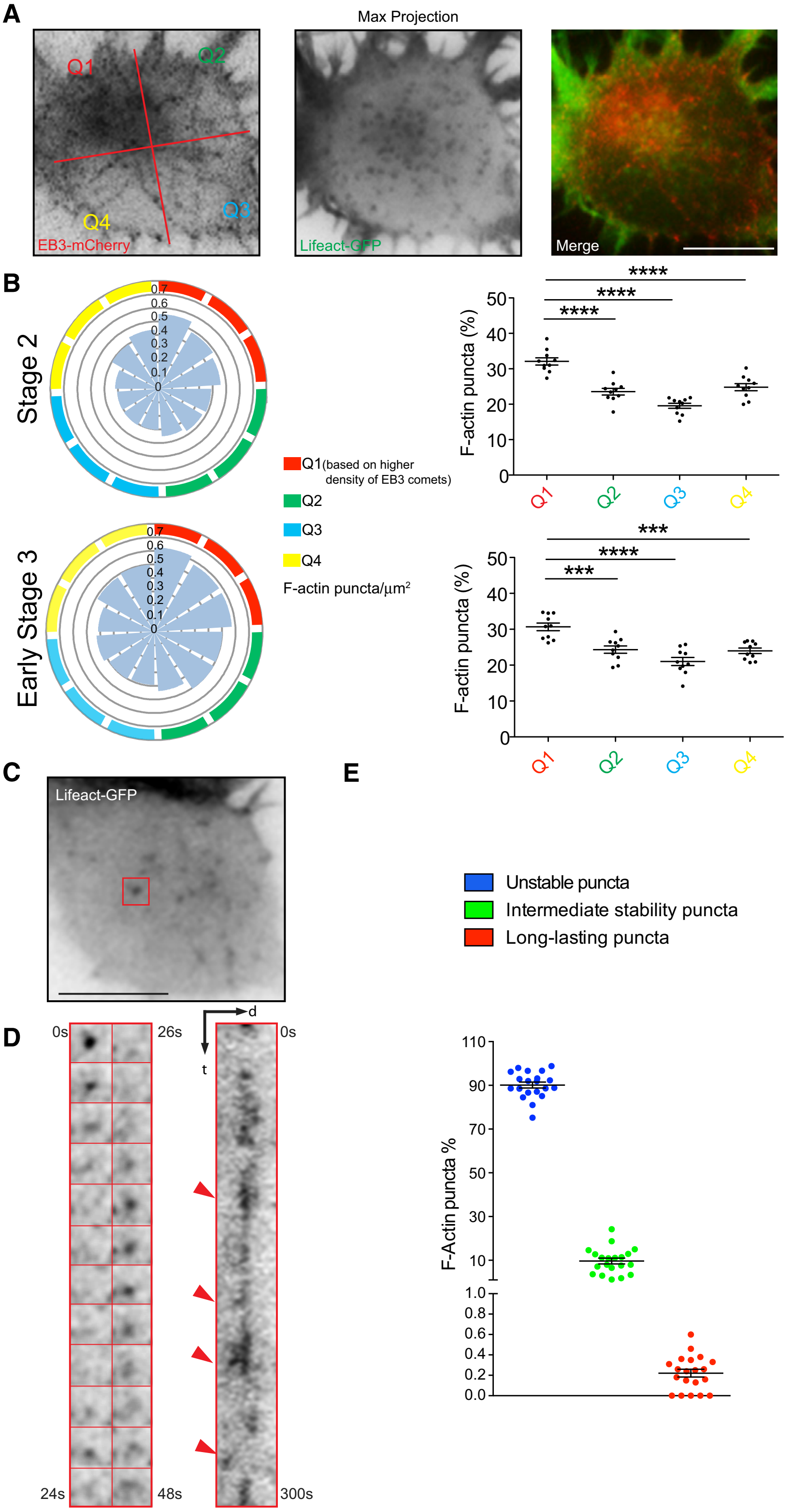


**Figure EV1. Dynamic F-actin puncta organized around the MTOC and show different blinking frequencies.**

- A Maximum projection of a time lapse of stage 1 neuron expressing EB3-mCherry and Lifeact-GFP. Higher density of F-actin puncta co-localizes in quadrant 1 (Q1) with more EB3 comets.
- B Left panel: distribution of F-actin puncta in the cell body from stage 2 cells and stage 3 cells. Right panel: percentage of F-actin puncta per quadrant during time-lapse analysis from stage 2 cells and stage 3 cells. The MTOC is always positioned at quadrant 1(Q1); $P < 0.0001$, all by one-way ANOVA, *post hoc* Dunnett test; ***$P < 0.001$, ****$P < 0.0001$. Mean ± SEM; $n = 10$ cells for each stage, from at least three different cultures.
- C Neuron (DIV1) transfected with Lifeact-GFP showing somatic F-actin puncta.
- D Montage and kymograph generated from the time lapse of cell shown in (C, region marked with red square) show dynamic F-actin punctum. Red arrowheads in the kymograph show comets generated from the blinking F-actin punctum.
- E Duration of F-actin puncta in the cell body of developing neurons. Unstable puncta (% in the somatic area) = 90.13 ± 1.33; intermediate stability puncta = 9.64 ± 1.31; long-lasting puncta = 0.22 ± 0.03. Mean ± SEM; $n = 20$ cells, from at least three different cultures.

Data information: Scale bar: 5 µm.

© 2019 The Authors

**Figure EV2. Somatic F-actin puncta act as rapid supply sources of F-actin to the periphery in developing neurons.**

A Lifeact-mEos3.2-expressing stage 2 neuron photoconverted in the soma with 405 nm laser (red circle with a diameter of 5.239 μm). Cell before (green signal) and after photoconversion (red signal).

B, C (B) Actin-mEos4b and (C) mEos3.2 expressing cells photoconverted in the soma using 405 nm laser (red circle with a diameter of 5.239 μm). Cell before and after photoconversion shows probe-specific behavior.

D Left panel: normalized intensities in the photoconverted area of Lifeact-mEos3.2, actin-mEos4b, or mEos3.2 expressing cells. Inset graph: Half-time ($t½$) values for Lifeact-mEos3.2 = 21.66 ± 1.937 ($n$ = 12), actin-mEos4b = 17.40 ± 1.275 ($n$ = 9), and mEos3.2 = 64.65 ± 9.205 ($n$ = 10). $P < 0.0001$ by one-way ANOVA, *post hoc* Dunnett's test, ***$P < 0.001$. Middle panel: photoconverted signal in neurite tips over time relative to the average initial signal from illuminated area for Lifeact-mEos3.2, actin-mEos4b, or mEos3.2 expressing cells. Right panel: Ratio of signal in neurite tip compared to soma upon photoconversion over time; all panels: mean ± SEM; $n$ = 12 for Lifeact-mEos3.2 cells, $n$ = 11 for mEos3.2 cells, and $n$ = 9 for actin-mEos4b cells.

E Neurites from cells in (A; inset 1), (B; inset 2), and (C; inset 3) show the reach of the photoactivated signal at the end of the time lapse (128–141 s).

F Normalized intensity values from the neurite tips plotted against their neurite lengths of Lifeact-mEos3.2, actin-mEos4b, and mEos3.2 expressing cells (from D) in the first 120 s after photoconversion.

Data information: Scale bar: 10 μm.



© 2019 The Authors

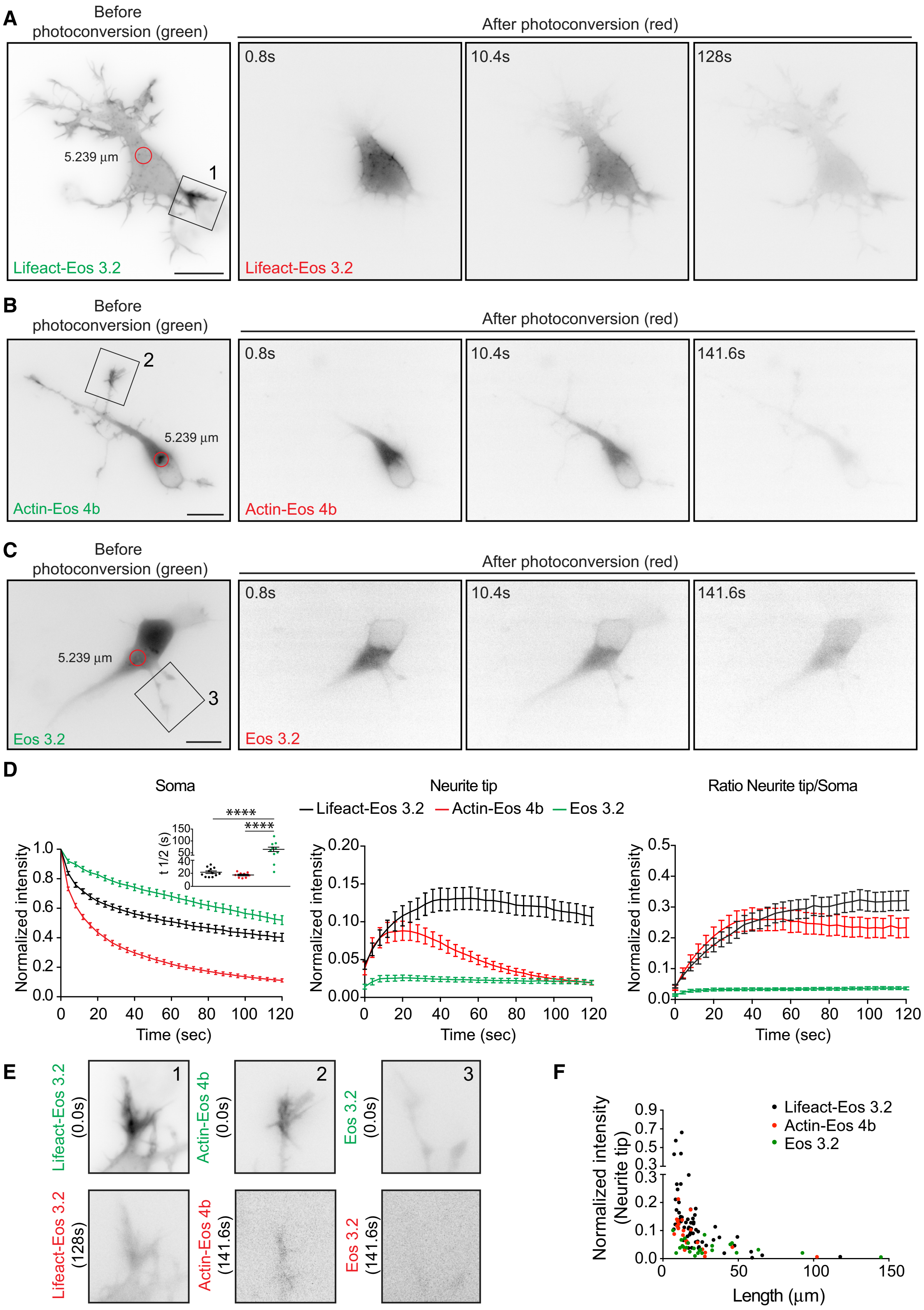
A
Before photoconversion (green)
After photoconversion (red)
0.8s
10.4s
128s
5.239 μm
1
Lifeact-Eos 3.2
Lifeact-Eos 3.2
B
Before photoconversion (green)
After photoconversion (red)
0.8s
10.4s
141.6s
2
5.239 μm
Actin-Eos 4b
Actin-Eos 4b
C
Before photoconversion (green)
After photoconversion (red)
0.8s
10.4s
141.6s
5.239 μm
3
Eos 3.2
Eos 3.2
D
Soma
Neurite tip
Ratio Neurite tip/Soma
Lifeact-Eos 3.2
Actin-Eos 4b
Eos 3.2
****
****
t 1/2 (s)
Normalized intensity
Time (sec)
E
Lifeact-Eos 3.2 (0.0s)
Actin-Eos 4b (0.0s)
Eos 3.2 (0.0s)
Lifeact-Eos 3.2 (128s)
Actin-Eos 4b (141.6s)
Eos 3.2 (141.6s)
F
Normalized intensity (Neurite tip)
Length (μm)
Lifeact-Eos 3.2
Actin-Eos 4b
Eos 3.2

**Figure EV2.**

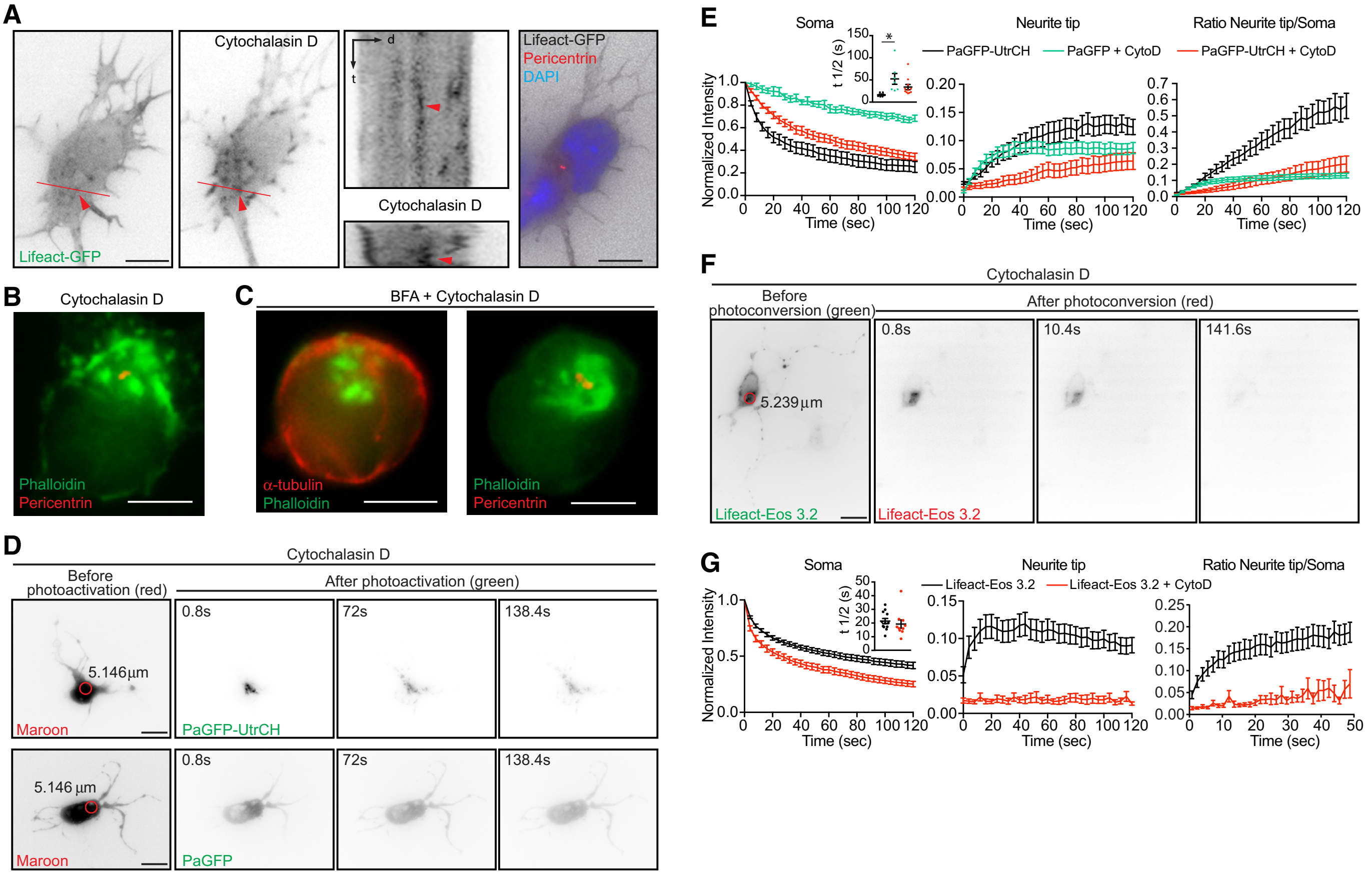


**Figure EV3. F-actin disruption affects release of somatic F-actin from the puncta to the periphery in developing neurons.**

A Time-lapse analysis revealed that F-actin cluster formation following cytochalasin D treatment originated from pre-existing intermittent F-actin puncta (seven cells from three different cultures).

B Cytochalasin D treatment produced F-actin clusters around the centrosome (83.44%; *n* = 157 from at least three different cultures).

C Brefeldin A (10 mg/ml for 12 h) treatment does not affect F-actin clusters after cytochalasin D treatment (95.24%; *n* = 21 cells from at least three different cultures).

D–G Cytochalasin D (1 µM for 45 min) precludes translocation of photoactivated PaGFP-UtrCH or photoconverted Lifeact-mEos3.2 signal from soma to the cell periphery. (D) PaGFP-UtrCH (upper panels) or PaGFP (lower panels) and mMaroon1 co-transfected neurons treated with cytochalasin D were illuminated in the soma using 405 nm laser (red circle with a diameter of 5.146 µm). After photoactivation the distribution of green signal shows how F-actin depolymerization affects PaGFP-UtrCH and PaGFP probes in distinct ways. (E) Left panel: normalized intensity values in the photoactivated area (soma) of untreated PaGFP-UtrCH, cytochalasin D-treated PaGFP-UtrCH,and PaGFP expressing cells. Inset graph: half-time ($t½$) values in seconds for untreated PaGFP-UtrCH cells = 16.29 ± 1.884 (*n* = 6), cytochalasin D-treated PaGFP cells = 52.31 ± 12.14 (*n* = 7), cytochalasin D-treated PaGFP-UtrCH cells = 33.92 ± 5.891 (*n* = 11). Mean ± SEM; *P* = 0.0259 by one-way ANOVA, *post hoc* Dunnett's test; $^*P < 0.05$. Middle panel: photoconverted signal in the neurite tip over time relative to the average initial signal from illuminated area for untreated PaGFP-UtrCH, cytochalasin D-treated PaGFP-UtrCH, and PaGFP expressing cells. Right panel: neurite tip to soma intensity ratio of photoactivated untreated PaGFP-UtrCH, cytochalasin D-treated PaGFP-UtrCH, and PaGFP expressing cells. All panels: mean ± SEM; *n* = 6 for untreated cells, *n* = 11 for cytochalasin D-treated PaGFP-UtrCH cells, *n* = 9 cells for cytochalasin D-treated PaGFP cells, from at least two different cultures. Experiments shown in Fig 5F and G, Fig EV3D and E and Appendix Fig S8C and D were done at the same time, therefore the same control data (Untreated PaGFP-UtrCH) is used for comparison. (F) Lifeact-mEos3.2 expressing cell treated with cytochalasin D and photoconverted in the soma with 405 nm laser (red circle with a diameter of 5.239 µm). Cell before (green) and after (red) photoconversion. (G) Left panel: normalized intensity values in the photoactivated area (soma) of untreated, cytochalasin D-treated Lifeact-Eos3.2 cells. Inset graph: half-time ($t½$) values in seconds for untreated cells = 21.44 ± 1.932 (*n* = 12), cytochalasin D-treated Lifeact-Eos3.2 cells = 19.26 ± 2.963 (*n* = 10). *P* = 0.5323 by unpaired Student's *t*-test. Middle panel: photoconverted signal in the neurite tip over time relative to the average initial signal from illuminated area for untreated and cytochalasin D-treated Lifeact-Eos3.2 cells. Right panel: neurite tip to soma intensity ratio of photoconverted signal in untreated, cytochalasin D-treated Lifeact-Eos3.2 cells. Experiments shown in Fig EV3F and G, Fig EV5E and F and Appendix Fig S8E and F were done at the same time, therefore the same control data (untreated Lifeact-Eos3.2) is used for comparison. All panels: mean ± SEM; *n* = 12 for untreated cells, *n* = 10 cells for cytochalasin D-treated cells from at least three different cultures.

Data information: Scale bar: 5 µm (A–C) and 10 µm (D and F).


© 2019 The Authors

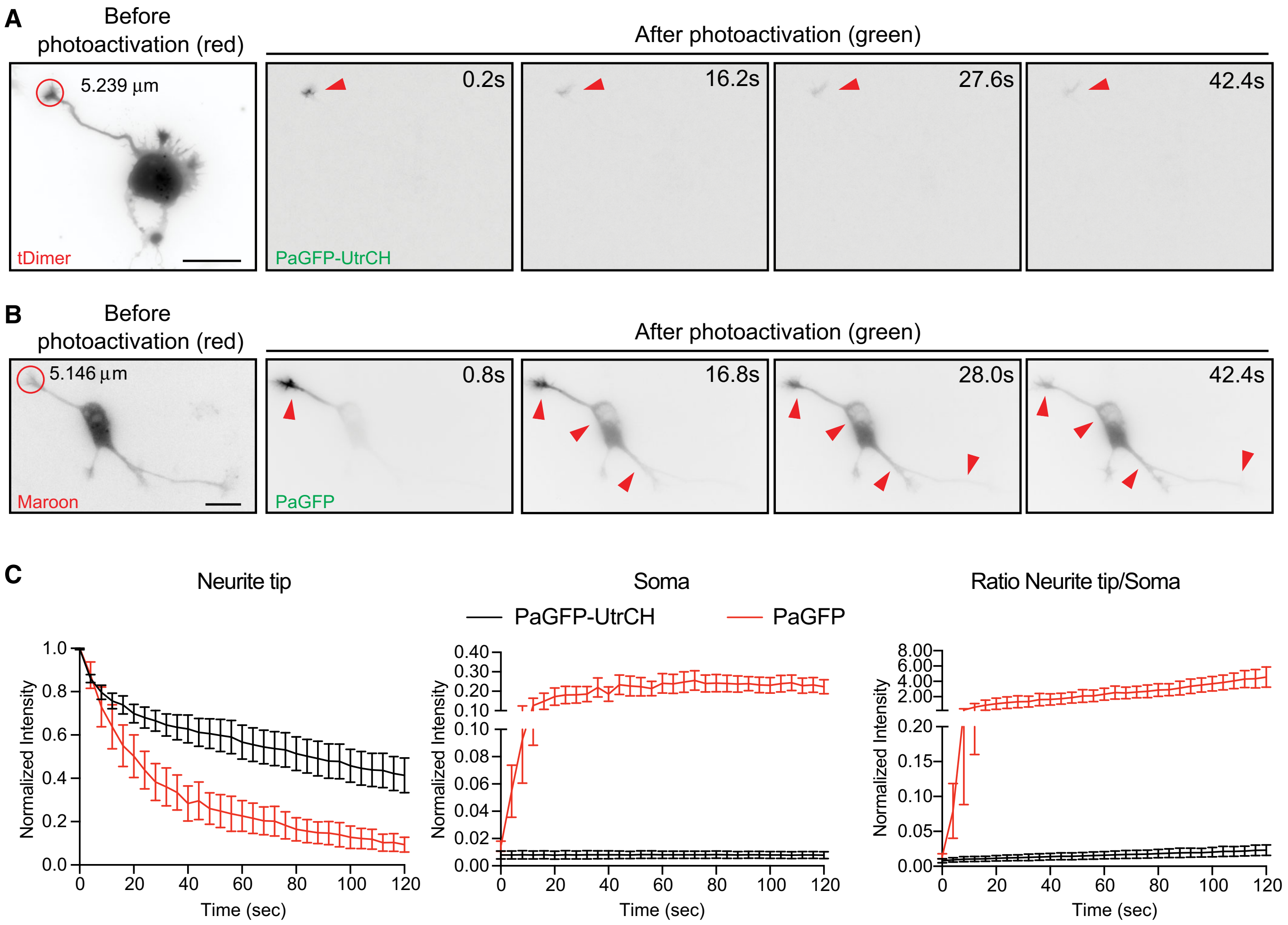


**Figure EV4. Photoactivated PaGFP-UtrCH in neurite tips does not translocate toward the cell body.**

A, B Neurons before (tDimer or mMaroon1 as indicated) and after photoactivation (green; PaGFP-UtrCH, A; PaGFP, B) at the neurite tip with 405 nm laser (red circle with a diameter of 5.239 µm in A, 5.146 µm in B). Red arrowheads point the reach of the photoactivated signal over time.

C Left panel: normalized intensity values in the photoactivated area (neurite tips) of PaGFP-UtrCH and PaGFP expressing cells. Middle panel: photoconverted signal in the soma over time relative to the average initial signal from illuminated area for PaGFP-UtrCH and PaGFP expressing cells. Right panel: neurite tip to soma intensity ratio of PaGFP-UtrCH and PaGFP signal. All panels: mean ± SEM; *n* = 8 cell for each group.

Data information: Scale bar: 10 µm.

**Figure EV5. CALI in somatic region distant from centrosome does not affect F-actin intensity and treadmilling in neurite tips.**

A Neurons transfected with Centrin2-KillerRed and EB3-GFP subjected to CALI. Left: Max projection of time lapse (40 s) before treatment; right: Max projection of time lapse (40 s) after treatment.

B Number of EB3 trajectories in the soma of example cell over time (before and after treatment). EB3 trajectories per $\mu m^2$ and minute compared by paired Student's *t*-test. Before CALI: 2.998 ± 0.279; after CALI: 2.171 ± 0.330; mean ± SEM; $n$ = 7 cells from three different cultures.

C Neurons transfected with Centrin2-KR and Lifeact-GFP were subjected to CALI in somatic regions distant from centrosome (control region). Left panel: Centrin2-KR signal before and after CALI; middle and right panels show Centrin2-KR labeling the centrosome before and after CALI and illuminated region marked as a red circle. Enlarged images of the neurite tips before (marked as 1) and after (marked as 2) CALI, red lines indicate the areas from where respective kymographs of actin treadmilling were obtained.

D Control irradiation does not affect F-actin treadmilling rate and F-actin intensity in neurite tips. Values are centered around the mean and expressed as standard deviations from the mean (*z*-score). Before Cali = −0.037 ± 0.165, After Cali = −0.215 ± 0.226. Mean ± SEM; $n$ = 10 cells from at least three different cultures; $P$ = 0.1522 by paired Student's *t*-test.

E Lifeact-mEos3.2 transfected DIV1 rat hippocampal neuron treated with nocodazole (7 μM for 1.5 h) photoactivated in the soma with 405 nm laser (red circle with a diameter of 5.239 μm). Cell before (green) and after (red) photoconversion. Arrowheads point photoconverted signal movement.

F Neurite tip to soma intensity ratio of photoconverted signal of untreated and nocodazole-treated Lifeact-mEos3.2 expressing cells. All panels: mean ± SEM; $n$ = 12 for untreated, $n$ = 7 for nocodazole-treated Lifeact-mEos3.2 expressing cells, from at least two different cultures.

Data information: Scale bar: 5 μm (A) 10 μm (C).

 © 2019 The Authors

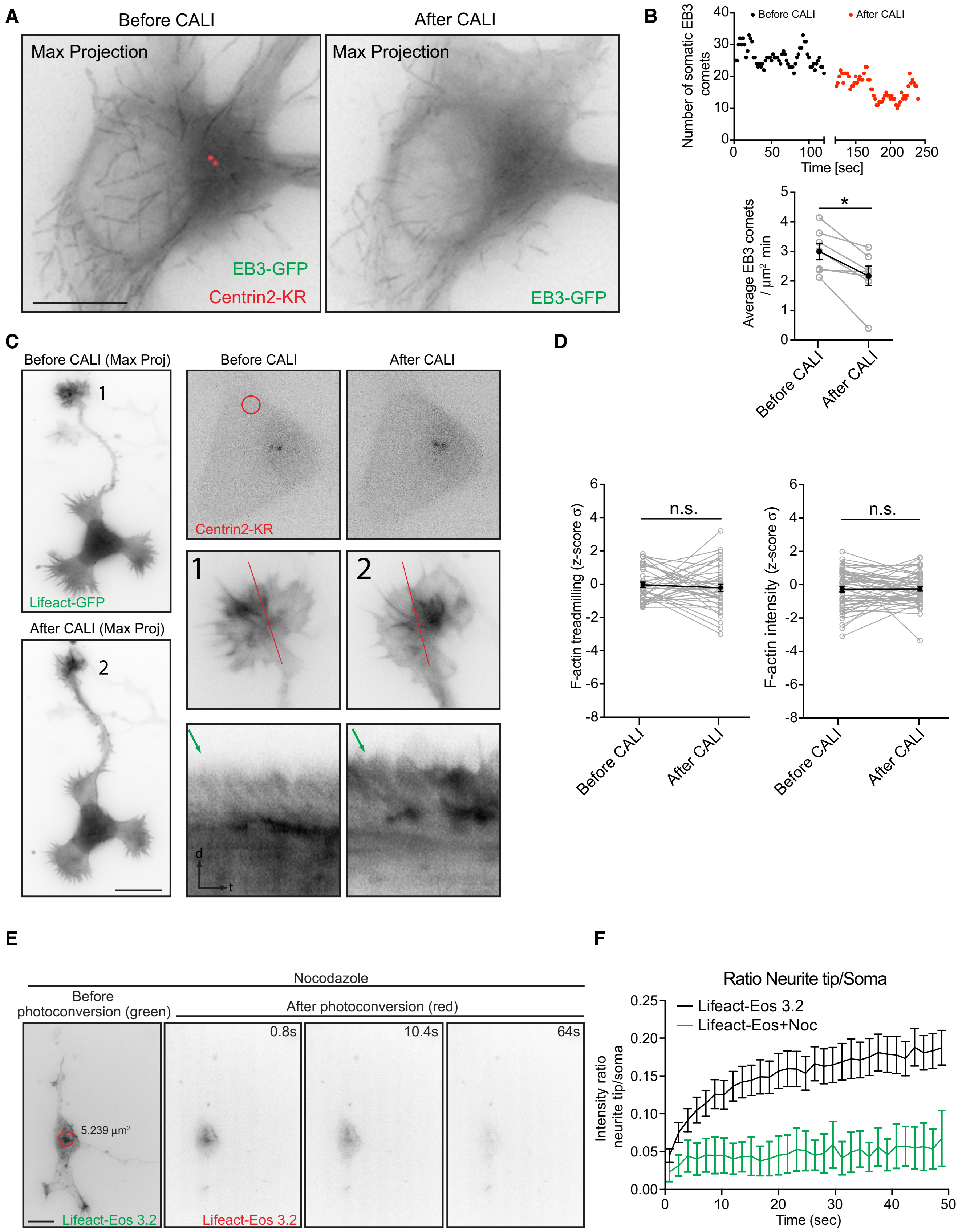
A
Before CALI
After CALI
Max Projection
Max Projection
EB3-GFP
Centrin2-KR
EB3-GFP
B
Before CALI
After CALI
Number of somatic EB3 comets
Time [sec]
Average EB3 comets / µm² min
*
Before CALI
After CALI
C
Before CALI (Max Proj)
Before CALI
After CALI
Centrin2-KR
Lifeact-GFP
After CALI (Max Proj)
d
t
D
n.s.
F-actin treadmilling (z-score σ)
Before CALI
After CALI
n.s.
F-actin intensity (z-score σ)
Before CALI
After CALI
E
Nocodazole
Before photoconversion (green)
After photoconversion (red)
0.8s
10.4s
64s
5.239 µm²
Lifeact-Eos 3.2
Lifeact-Eos 3.2
F
Ratio Neurite tip/Soma
Lifeact-Eos 3.2
Lifeact-Eos+Noc
Intensity ratio neurite tip/soma
Time (sec)

**Figure EV5.**
