# Supplementary material for: Radial somatic F‐actin organization affects growth cone dynamics during early neuronal development
Source: EMBO Rep. 2019 Oct 24;20(12):e47743. doi: 10.15252/embr.201947743 (PMC6893363; doi:10.15252/embr.201947743)
Supplement: Supplementary file 3 — Movie EV1 [file EMBR-20-e47743-s003.zip › Movie_EV1.docx]

**Movie EV1.**

**Time-lapse imaging of a somatic F-actin punctum incubated with 250nM SiR-actin.** STED imaging was performed on a Leica TCS SP8 gated STED microscope equipped with a pulsed 775 nm depletion laser (80 MHz) and a pulsed white light laser (WLL) for excitation. Duration of time-lapse imaging: 34 sec. Interval between the frames is 1.7 sec.
