# Supplementary material for: Radial somatic F‐actin organization affects growth cone dynamics during early neuronal development
Source: EMBO Rep. 2019 Oct 24;20(12):e47743. doi: 10.15252/embr.201947743 (PMC6893363; doi:10.15252/embr.201947743)
Supplement: Supplementary file 5 — Movie EV3 [file EMBR-20-e47743-s005.zip › Movie_EV3.docx]

**Movie EV3.**

**Photoconversion in the soma of stage 2 neurons expressing Lifeact-mEos3.2.**

Imaging was performed on a Visitron Systems VisiScope TIRF/FRAP imaging system based on a Nikon Ti-E equipped with a Nikon CFI Apo TIRF 100x, 1.49 NA oil objective. 405 nm laser illumination is performed in a circular region with a dimeter of 7.182 µm to achieve photoconversion in the soma. Duration of time-lapse imaging: 144 sec; 2.4 sec before and 141.6 sec after photoconversion. Interval between the frames is 0.8 sec.
