# Supplementary material for: Radial somatic F‐actin organization affects growth cone dynamics during early neuronal development
Source: EMBO Rep. 2019 Oct 24;20(12):e47743. doi: 10.15252/embr.201947743 (PMC6893363; doi:10.15252/embr.201947743)
Supplement: Supplementary file 7 — Movie EV5 [file EMBR-20-e47743-s007.zip › Movie_EV5.docx]

**Movie EV5.**

**Photoactivation in the soma of stage 2 neurons expressing PaGFP-UtrCH or PaGFP and tDimer treated with 1µM cytochalasin D.**

Imaging was performed on a Visitron Systems VisiScope TIRF/FRAP imaging system based on a Nikon Ti-E equipped with a Nikon CFI Apo TIRF 100x, 1.49 NA oil objective. 405 nm laser illumination is performed in a circular region with a dimeter of 5.146 µm to achieve photoactivation in the somas of the cells. Duration of time-lapse imaging: 137 sec; 2.4 sec before and 134.6 sec after photoactivation. Interval between the frames is 0.8 sec.
