# Supplementary material for: Radial somatic F‐actin organization affects growth cone dynamics during early neuronal development
Source: EMBO Rep. 2019 Oct 24;20(12):e47743. doi: 10.15252/embr.201947743 (PMC6893363; doi:10.15252/embr.201947743)
Supplement: Supplementary file 8 — Movie EV6 [file EMBR-20-e47743-s008.zip › Movie_EV6.docx]

**Movie EV6.**

**Photoactivation in the growth cone of stage 2 neuron expressing PaGFP and mMaroon1.** Imaging was performed on a Visitron Systems VisiScope TIRF/FRAP imaging system based on a Nikon Ti-E equipped with a Nikon CFI Apo TIRF 100x, 1.49 NA oil objective. 405 nm laser illumination is performed in a circular region with a dimeter of 5.146 µm to achieve photoactivation in the growth cone. Duration of time-lapse imaging: 137 sec; 2.4 sec before and 134.6 sec after photoactivation. Interval between the frames is 0.8 sec.
