# Supplementary material for: Radial somatic F‐actin organization affects growth cone dynamics during early neuronal development
Source: EMBO Rep. 2019 Oct 24;20(12):e47743. doi: 10.15252/embr.201947743 (PMC6893363; doi:10.15252/embr.201947743)
Supplement: Supplementary file 9 — Movie EV7 [file EMBR-20-e47743-s009.zip › Movie_EV7.docx]

**Movie EV7.**

**Photoactivation in the growth cone of stage 2 neuron expressing PaGFP-UtrCH and tDimer.**

Imaging was performed on a Visitron Systems VisiScope TIRF/FRAP imaging system based on a Nikon Ti-E equipped with a Nikon CFI Apo TIRF 100x, 1.49 NA oil objective. 405 nm laser illumination is performed in a circular region with a dimeter of 5.239 µm to achieve photoactivation in the soma. Duration of time-lapse imaging: 98 sec; 0.6 sec before and 95.6 sec after photoactivation. Interval between the frames is 0.2 sec.
