# Supplementary material for: Radial somatic F‐actin organization affects growth cone dynamics during early neuronal development
Source: EMBO Rep. 2019 Oct 24;20(12):e47743. doi: 10.15252/embr.201947743 (PMC6893363; doi:10.15252/embr.201947743)
Supplement: Supplementary file 10 — Movie EV8 [file EMBR-20-e47743-s010.zip › Movie_EV8.docx]

**Movie EV8.**

**Time-lapse imaging of neurons expressing Lifeact-RFP and Drebrin-YFP or Drebrin S142D-YFP.**

Epi-fluorescence imaging was performed on an inverted Nikon microscope (Eclipse, Ti) with a 60x objective (NA 1.4). Duration of time-lapse imaging: 5 min. Interval between the frames is 2 sec.
