# Supplementary material for: Radial somatic F‐actin organization affects growth cone dynamics during early neuronal development
Source: EMBO Rep. 2019 Oct 24;20(12):e47743. doi: 10.15252/embr.201947743 (PMC6893363; doi:10.15252/embr.201947743)
Supplement: Supplementary file 13 — Movie EV11 [file EMBR-20-e47743-s013.zip › Movie_EV11.docx]

**Movie EV11.**

**CALI of somatic region outside of the centrosome in early stage 3 neuron expressing Centrin2-KR and Lifeact-GFP.**

Cell was imaged for 5 min (interval between the frames is 2 sec) before laser irradiation (Before CALI). 2-3 hrs. after irradiation (After CALI) the same cell was imaged 5 min (interval between the frames is 2 sec). Epi-fluorescence imaging was performed on an inverted Nikon microscope (Eclipse, Ti) with a 100x objective (NA 1.49).
